# Altered gut microbiota in temporal lobe epilepsy with anxiety disorders

**DOI:** 10.3389/fmicb.2023.1165787

**Published:** 2023-05-22

**Authors:** Shouchao Wei, Yingren Mai, Li Hu, Ruxing Zheng, Dongming Zheng, Wenrong Chen, Yan Cai, Junjun Wang

**Affiliations:** ^1^Department of Neurology, Central People's Hospital of Zhanjiang, Zhanjiang, China; ^2^Zhanjiang Institute of Clinical Medicine, Central People's Hospital of Zhanjiang, Zhanjiang, China; ^3^Department of Neurology, The Second Affiliated Hospital of Guangzhou Medical University, Zhanjiang, China; ^4^Department of Histology and Embryology, Guangdong Medical University, Zhanjiang, China; ^5^Jiangsu Key Laboratory of Neuropsychiatric Diseases and Institute of Neuroscience, Soochow University, Suzhou, China

**Keywords:** temporal lobe epilepsy patient, anxiety disorder, gut microbiota, bacteria, fungi

## Abstract

**Introduction:**

Patients with epilepsy are particularly vulnerable to the negative effects of anxiety disorders. In particular, temporal lobe epilepsy with anxiety disorders (TLEA) has attracted more attention in epilepsy research. The link between intestinal dysbiosis and TLEA has not been established yet. To gain deeper insight into the link between gut microbiota dysbiosis and factors affecting TLEA, the composition of the gut microbiome, including bacteria and fungi, has been examined.

**Methods:**

The gut microbiota from 51 temporal lobe epilepsy patients has been subjected to sequencing targeting 16S rDNA (Illumina MiSeq) and from 45 temporal lobe epilepsy patients targeting the ITS-1 region (through pyrosequencing). A differential analysis has been conducted on the gut microbiota from the phylum to the genus level.

**Results:**

TLEA patients' gut bacteria and fungal microbiota exhibited distinct characteristics and diversity as evidenced by high-throughput sequencing (HTS). TLEA patients showed higher abundances of *Escherichia*-*Shigella* (genus), Enterobacterales (order), Enterobacteriaceae (family), Proteobacteria (phylum), Gammaproteobacteria (class), and lower abundances of Clostridia (class), Firmicutes, Lachnospiraceae (family), Lachnospirales (order), and *Ruminococcus* (genus). Among fungi, *Saccharomycetales fam*. *incertae sedis* (family), *Saccharomycetales* (order), *Saccharomycetes* (class), and *Ascomycota* (phylum) were significantly more abundant in TLEA patients than in patients with temporal lobe epilepsy but without anxiety. Adoption and perception of seizure control significantly affected TLEA bacterial community structure, while yearly hospitalization frequency affected fungal community structures in TLEA patients.

**Conclusion:**

Here, our study validated the gut microbiota dysbiosis of TLEA. Moreover, the pioneering study of bacterial and fungal microbiota profiles will help in understanding the course of TLEA and drive us toward preventing TLEA gut microbiota dysbiosis.

## Introduction

Epilepsy is a chronic disease characterized by sudden, short-lived, and recurrent central nervous system dysfunction caused by abnormal discharge of brain neurons (Christensen et al., 2022). Temporal lobe epilepsy, one of the most common types of epilepsy, affects ~65 million people worldwide (Beghi, 2020; Buchin et al., 2022). Epilepsy patients frequently experience more psychological pressure and are more prone to mental illness than the general population (Tang et al., 2022). Up to 60% of epilepsy patients experience anxiety and/or depression (Seid et al., 2022).

Numerous studies have confirmed the alteration of enterobacterial structure in patients with neuropsychiatric and neurodegenerative disorders, whereas few studies have assessed the potential associations of epilepsy and intestinal fungi (Ding et al., 2021; Fusco et al., 2022; Iannone et al., 2022). Specifically, only a few population-based studies have confirmed gut flora dysbiosis in epilepsy patients. The sample size is relatively limited, so the results could be inconsistent (Ding et al., 2021). A study from Western China (Guizhou province) showed that *Fusobacterium* sp., *Fusobacterium mortiferum, Ruminococcus gnavus*, and *Bacteroides fragilis* were significantly positively correlated with the occurrence of epilepsy (*r* ≥ 0.5, *P* < 0.05) (Dong et al., 2022). Another study also from Western China (Sichuan Province) showed *Actinomyces, Verrucomicrobia, Nitrospirae* and *Blautia, Bifidobacterium, Subdoligranulum, Dialister*, and *Anaerostipes* were predominantly found in the intestines of patients with anti-seizure medications (Kruskal–Wallis test, *P* < 0.05) (Gong et al., 2020). Although most epilepsy patients are from Western China, their intestinal flora structure is different. We hypothesized that different types of epilepsy have different prognoses and effects on the intestinal flora. Therefore, it is necessary to determine the type of epilepsy before performing intestinal flora analysis.

As defined for epilepsy patients in the Diagnostic and Statistical Manual of Mental Disorders, anxiety is one of the most common psychiatric comorbidities as well as one of the most complex ones exhibiting a wide spectrum of manifestations from paroxysmal symptoms to epilepsy-specific anxiety and classic anxiety disorders (Munger Clary, 2022). However, the specific mechanisms underlying the comorbidity of epilepsy and anxiety are so far unclear. The neurotransmitters such as 5-hydroxytryptamine, gamma-aminobutyric acid (GABA), norepinephrine, and dopamine have been reported to play a crucial role in the pathogenesis of temporal lobe epilepsy with anxiety disorders (TLEA) (Nutt et al., 2022; Xu et al., 2022).

Given the role of gut microbiota in mental health, breakthroughs happened in the last decade. We note that animal experiments and clinical trials have shown the beneficial effects of probiotics such as *Lactobacillus R0052* and *Bifidobacterium longum R0175* on anxiety (Mitrea et al., 2022). This cumulative evidence points to the vital role of gut microbes in anxiety disorders. Regrettably, the gut microbiota structure and function in TLEA patients and how these differ from those in epilepsy individuals are still not fully explored.

In this study, the composition of the gut microbiota, including bacteria and fungi, has been assessed and thereby identified potent microecological biomarkers of the disease. Despite the small cohort size, this is the first study comparing the gut bacterial and fungal flora of individuals with TLEA and patients with temporal lobe epilepsy but without anxiety disorder (TLEW).

## Materials and methods

The temporal lobe epilepsy patients at the Department of Neurology and the Neuroelectrophysiology Department of the Central People's Hospital of Zhanjiang (CPHZ) have been recruited for this study. Temporal lobe epilepsy was diagnosed by using an electroencephalogram (EEG) based on observed or reported seizures and interictal/seizure phases, epileptic focal localization, patients' clinical presentations, imaging, and EEG localizing the epileptogenic zone in the ipsilateral temporal lobe. The food intake of each patient was normal during the week before enrollment, and all patients included in the study had a balanced diet.

In the following instances, individuals were excluded: (1) if patients had received anti-seizure medication treatment, such as anti-anxiety and depression medications, antidiarrheal medications, laxatives, and antibiotics, or probiotic supplements 1 month before enrollment; (2) if they had undergone bowel resection or experienced acute gastric intestinal bleeding or intestinal tumors; (3) if they had undergone gastroscopy, colonoscopy, gastrointestinal barium meal examination, or other invasive digestive system examinations within 6 months and had a history of digestive system-related surgery; (4) if they were suffering from inflammatory disease enteropathy, Crohn's disease, or other digestive system diseases; (5) if they had diseases that may affect the stability of the intestinal flora due to coagulation dysfunction, severe cardiopulmonary disease, hypertension, diabetes, digestive system, immune system, and others; and (6) if they had a history of alcoholism, alcohol dependence, and smoking.

### Clinical assessment

**Figure 1:**
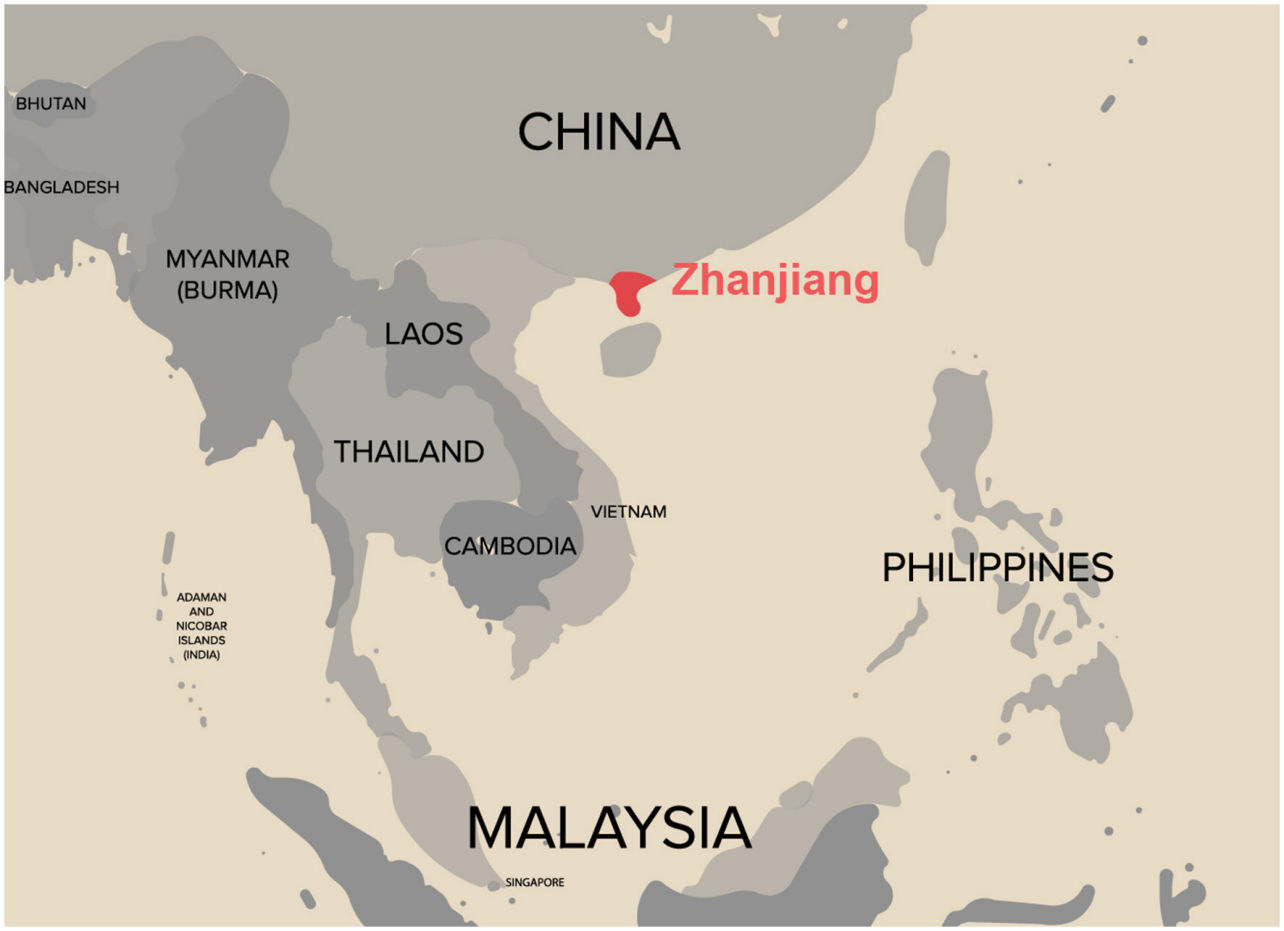
Geographic distribution of the study population. Only Han Chinese individuals residing in populations in tropical China were included, thus mitigating the effect of ethnic and geographic bias.

**Figure 2:**
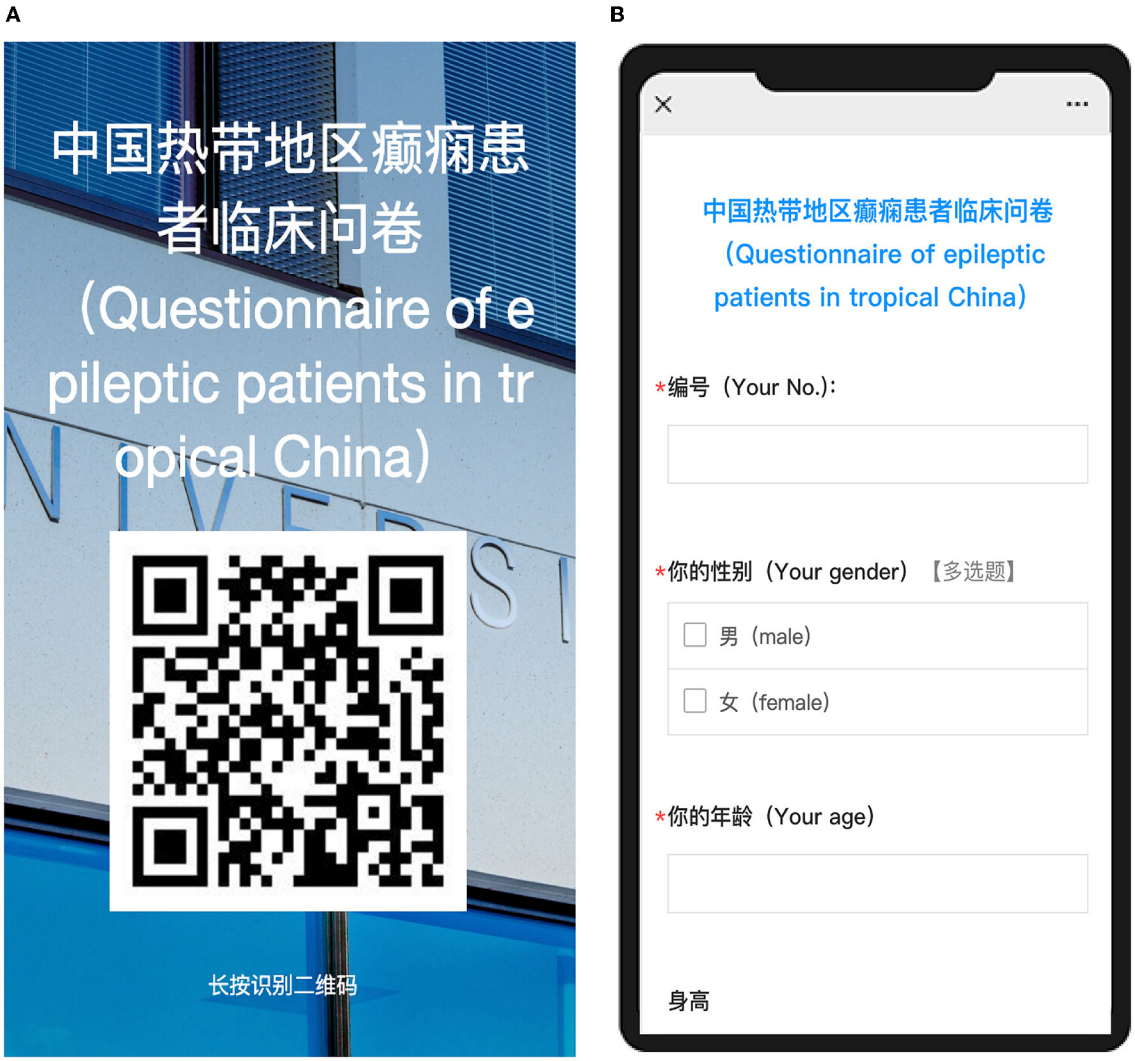
Anonymous questionnaire for collecting demographic characteristics, disease characteristics, and treatment characteristics. **(A)** QR code for logging in to the anonymous questionnaire; **(B)** the user interface of the anonymous questionnaire.

All temporal lobe epilepsy patients were recruited consecutively between December 2021 and December 2022. Patients were between the ages of 18 and 48 years and were of Han Chinese descent, residing in tropical China (Figure 1). The presence or absence of concomitant anxiety in patients with temporal lobe epilepsy served as the basis to establish TLEA and TLEW groups. Information on each participant's gender, epilepsy disease characteristics, and use of medication was recorded as demographic (age, gender, and education) and anxiety impact factors (AIFs, including the type of epilepsy, age at onset, duration of epilepsy, seizure frequency, and seizure type), to identify key factors influencing the presence of anxiety manifestations in patients with epilepsy. Questionnaires were produced using the Questionnaire Star tool (Figures 2A, B).

In this study, anxiety has been defined utilizing the Hamilton Anxiety Rating Scale (Ham-A), generalized anxiety disorder 7 (GAD-7), and self-rating anxiety scale (SAS) scores, with higher scores indicating higher levels of anxiety. The Ham-A score for TLEA was ≥14 and that for TLEW was <7. The GAD-7 TLEA score was >5. According to the Chinese normative results, the cutoff value of the SAS standard score was 50; thus, the TLEA score was >50.

All scores were independently evaluated by clinicians who were blind concerning the patients' clinical conditions and who had attended a training course on how to administer the tests before the study. All patients considered to suffer from a psychiatric disorder or to be in clinical need of psychiatric help were offered assistance by a psychiatrist or psychotherapist. The Clinical Trials Ethics Committee CPHZ (approval number: PJI117-2022-027-013) approved the study protocols, and all methods followed the principles outlined in the 1975 Declaration of Helsinki. All participants participated voluntarily and provided written informed consent. The personal privacy of each participant was protected.

### Sample collection and other: DNA extraction and sequencing

Sterile plastic cups were provided to each participant to collect a fresh fecal sample in the morning. All samples were transported to the Institute of Clinical Medicine affiliated with the CPHZ in a transfer box containing ice packs, and samples were kept at −80°C for 1 h after collection. These samples have been transported to Major-Bio (Shanghai, China) for DNA extraction and sequencing.

The microbial DNA from 51 samples has been extracted utilizing the E.Z.N.A.^®^ Soil DNA Kit (Omega Bio-Tek, Norcross, GA, United States), following the manufacturer's instructions. A final DNA concentration, as well as purity, was assessed using a NanoDrop 2000 UV-VIS spectrophotometer (Thermo Scientific, Wilmington, DE, United States), and the quality of DNA was determined through 1% agarose gel electrophoresis. For amplifying the bacterial 16S rRNA in the V3–V4 region, the polymerase chain reaction (PCR) has been performed using 338F (ACTCCTACGGGAGGCAGCAG) and 806R (ACTCCTACGGGAGGCAGCAG) primers with Trans Start Fast pfu DNA polymerase (Trans Gen, Beijing, China) in an ABI Gene Amp 9700 device (Applied Biosystems, CA, United States). For amplifying the ITS-1 fragment of fungi, ITS1F (CTTGGTCATTTAGAGGAAGTAA) and ITS2R (GCTGCGTTCTTCATCGATGC) primers were utilized. PCR products were excised from 2% agarose gels, further purified using an AxyPrep DNA Gel Extraction Kit (Axygen Biosciences, Union City, CA, United States), and quantified using QuantiFluor™-ST (Promega, Madison, WI, United States).

### Sequencing and data analyses

Purified amplicons were pooled in equimolar amounts, following the standard protocol of Bio-Pharm Technology Co. Ltd. at Majorbio (Shanghai, China), by demultiplexing, quality-filtering [using Trimmomatic software (V0.36, http://www.usadellab.org/cms/?page=trimmomatic)], and merging [with Flash software (V 1.2.11, https://ccb.jhu.edu/software/FLASH/index.shtml)], and raw FastQ files were further processed. The datasets presented in this study can be found in online repositories (Bio Project ID: PRJNA934740).

Abundances of operational taxonomic units (OTUs) were subjected to normalization according to the minimum sequence of the sample using standard sequence numbers and thereby clustered at 97% similarity using UPARSE (V 11, http://drive5.com/uparse). UCHIME was used to identify and remove chimeric sequences. Rare taxa were <10 OTUs (Wasserstrom et al., 2017). A classification analysis has been conducted utilizing the 16S rRNA SILVA database (SSU132) and a fungal database (Unite 8.0) with a 70% confidence threshold.

The data analyses were conducted utilizing an open online platform of the Majorbio cloud platform (https://cloud.majorbio.com/) (Ren et al., 2022). QIIME (V1.9.1, http://qiime.org/install/index.html) has been applied for determining α- as well as β-diversities and also for both principal component analysis (PCA) and principal coordinate analysis (PCoA). Bar diagrams of the microbial community were used to show the community structure composed of different groups at various taxonomic levels (Li et al., 2018). To search for statistically different biomarkers of TLEA, linear discriminant analysis (LDA) and effect size measurements (LEfSe) were performed using the LEfSe tool. The similarity test (ANOSIM) of the PRIMER 6 software package (PRIMER-E Ltd., Luton, UK) has been utilized to assess the fecal flora variations between the TLEA and TLEW groups. Identifying the distinct functional groups and relating corresponding abundances among bacterial as well as fungal communities have been evaluated utilizing PICRUSt 2 software (V2.2.0, https://github.com/picrust/picrust2/) and the Kyoto Encyclopedia of Genes and Genomes (KEGG) database (bacteria) and an open annotation tool, FunGuild (V1, http://www.funguild.org/), Fungi Functional Guild (fungi). The composition of TLEA bacterial and fungi communities and their potential correlations with AIFs have been determined by redundancy analysis (RDA) using R software (V3.3.1, https://www.r-project.org/).

### Statistical analyses

Bacteria, as well as fungi, have been evaluated at phylum, class, order, family, and genus levels, while the data series were analyzed using several different scales. Subgroup analyses were performed based on demographic and treatment regimens. The SPSS statistical package (V20.0) (IBM, Armonk, NY, United States) was applied to analyze the baseline data. The results of the measurement data were analyzed to identify differences between groups using *t*-tests and Wilcoxon rank-sum tests, whereas differences between groups in count data were analyzed using chi-square tests. Statistical significance is reported at the criterion of *P* < 0.05, and the *P*-value was adjusted by the false discovery rate (FDR) on the Majorbio cloud platform.

## Results

### Demographic characteristics of TLEA and TLEW patients

A total of 51 and 45 temporal lobe epilepsy patients provided stool samples for gut bacteria and gut fungi analyses, respectively. All participants were from a tropical Chinese population (Figure 1) and were of Han ethnicity. The demographic and epileptic disease features of the subjects are presented in Table 1 and Supplementary Table 2. The lifestyle and clinical data of all participants were recorded. TLEA and TLEW patients did not vary considerably in terms of age, gender, and body mass index (Table 1). However, TLEA patients showed significantly higher Ham-A (*P* < 0.05), SAS (*P* < 0.05), and GAD-7 (*P* < 0.05) scores. Analysis of the AIFs that may affect the occurrence of anxiety among patients with epilepsy revealed significant differences in the TLEA group regarding employment, working strength, distance to hospital, annual hospitalization frequency, treatment regimen, disease duration, and perception of seizure control.

**Table 1:** Characteristics of 51 temporal lobe epilepsy patients^*^.

**Characteristics**	**Statistics**	**TLEA, *n* = 27**	**TLEW, *n* = 24**		***P***
Age	Yrs, mean ± SD	29.3 ± 5.7	31.3 ± 7.2	1.108	0.273
BMI	Kg/m^2^, mean ± SD	23.6 ± 1.9	22.3 ±2.5	1.093	0.280
SAS	Mean ± SD	60.2 ± 3.2	27.5 ± 3.0	−37.581	0.000
GAF-7	Mean ± SD	12.2 ± 1.5	2.0 ± 1.1	−26.485	0.000
HAMA	Mean ± SD	22.0 ± 4.3	5.5 ± 1.1	−18.422	0.000
Gender	Female	16 (59.2)	10 (41.7)	0.004	0.974
	Male	11 (40.7)	14 (58.3)		
Marital status	Married	17 (63.0)	14 (58.3)	0.114	0.735
	Unmarried or other marital status	10 (37.0)	10 (41.7)		
Education	Primary (0–9 years)	5 (18.5)	8 (33.3)	1.833	0.400
	Secondary (9–12 years)	16 (59.3)	13 (54.2)		
	Higher (12 years)	6 (22.2)	3 (1.3)		
Employment	Employed (or student)	14 (51.9)	20 (83.3)	5.667	0.017
	Unemployed	13 (48.1)	4 (16.7)		
Working strength	Less activity (office, and so on)	8 (29.6)	8 (33.3)	8.717	0.013
	Light-to-moderate activity (installers and so on)	9 (33.3)	15 (62.5)		
	Moderate or heavy activity (agriculture and so on)	10 (37.0)	1 (4.2)		
Capital income per month (RMB)	<2,000	7 (25.9)	8 (33.3)	0.522	0.914
	2,000–4,999	14 (51.9)	12 (50)		
	5,000–9,999	4 (14.8)	3 (12.5)		
	>10,000	2 (7.4)	1 (4.2)		
Type of insurance	Rural cooperative medical care	10 (37.0)	15 (62.5)	3.302	0.192
	Urban medical insurance	13 (48.1)	7 (29.2)		
	None	4 (14.8)	2 (8.3)		
Distance to hospital (km)	≥10	21 (77.8)	12 (50)	4.293	0.038
	<10	6 (22.2)	12 (50)		
Hospitalization frequency yearly	0	4 (14.8)	3 (12.5)	10.135	0.006
	1–4	21 (77.8)	11 (45.8)		
	≥5	2 (7.4)	10 (41.7)		
Medical care	Very good/good	14 (51.9)	18 (75)	2.913	0.088
	Bad/no idea	13 (48.1)	6 (25)		
Beyond annual household income^**^	Yes	12 (44.4)	15 (62.5)	1.057	0.304
	No	15 (55.6)	9 (37.5)		
Convenience of appointment	Yes	16 (59.3)	11 (45.8)	0.919	0.338
	No/sometimes	11 (40.7)	13 (54.2)		
Epilepsy syndrome	Symptomatic focal epilepsy	13 (48.1)	16 (66.7)	1.776	0.183
	Cryptogenic generalized	14 (51.9)	8 (33.3)		
Treatment regimen	Monotherapy	12 (44.4)	19 (79.2)	6.426	0.011
	Polytherapy	15 (55.6)	5 (20.8)		
Seizures in the past 30 days	Controlled	11 (40.7)	14 (58.3)	1.574	0.210
	Uncontrolled	16 (59.3)	10 (41.7)		
Current antiepileptic drugs	Carbamazepine	7	12	3.150	0.076
	Oxasipine	11	11	0.134	0.714
	Topiramate	8	6	0.137	0.712
	Sodium valproate	9	3	3.065	0.080
	Pirampanide	4	1	1.629	0.202
	Chinese Medicine	11	9	0.056	0.813
	Others	8	6	0.137	0.712
Disease duration (years)	<0.5	6 (22.2)	12 (50)	6.482	0.039
	0.5–2	5 (18.5)	6 (25)		
	>2	16 (59.2)	6 (25)		
Daily dosing frequency	<1	1 (3.7)	4 (16.7)	5.433	0.246
	1	4 (14.8)	3 (12.5)		
	2	1 (3.7)	2 (8.3)		
	3	10 (37.0)	11 (45.8)		
	>3	11 (40.7)	4 (16.7)		
Perception of seizure control	Controlled	6 (22.2)	14 (58.3)	6.951	0.008
	Uncontrolled or not always controlled	21 (77.8)	10 (41.7)		
Care from family or friends	Continuous/almost	24 (88.9)	23 (95.8)	0.848	0.357
	Rare/absent/never	3 (11.1)	1 (4.2)		

^*^Categorical variables were summarized according to the absolute frequency and percentage of subjects (%) in each category level.

^**^Beyond annual household income, the health care costs exceed the average annual household income.

TLEA, temporal lobe epilepsy with anxiety disorders; TLEW, temporal lobe epilepsy but without anxiety disorder; SAS, Self-Rating Anxiety Scale; GAD, Generalized Anxiety Disorder; HAMA, Hamilton Anxiety Scale.

### Lower gut bacteria diversity in TLEA patients

16S sRNA gene sequencing has been applied for analyzing the bacteria fractions in the samples as well as for assessing the degree of bacterial flora dysbiosis associated with TLEA. Coverage in all samples was estimated at >99.9%. After removing rare OTUs, 11 phyla, 16 classes, 38 orders, 75 families, 208 genera, 404 species, and 598 OTUs have been retained to carry out further analyses.

**Figure 3:**
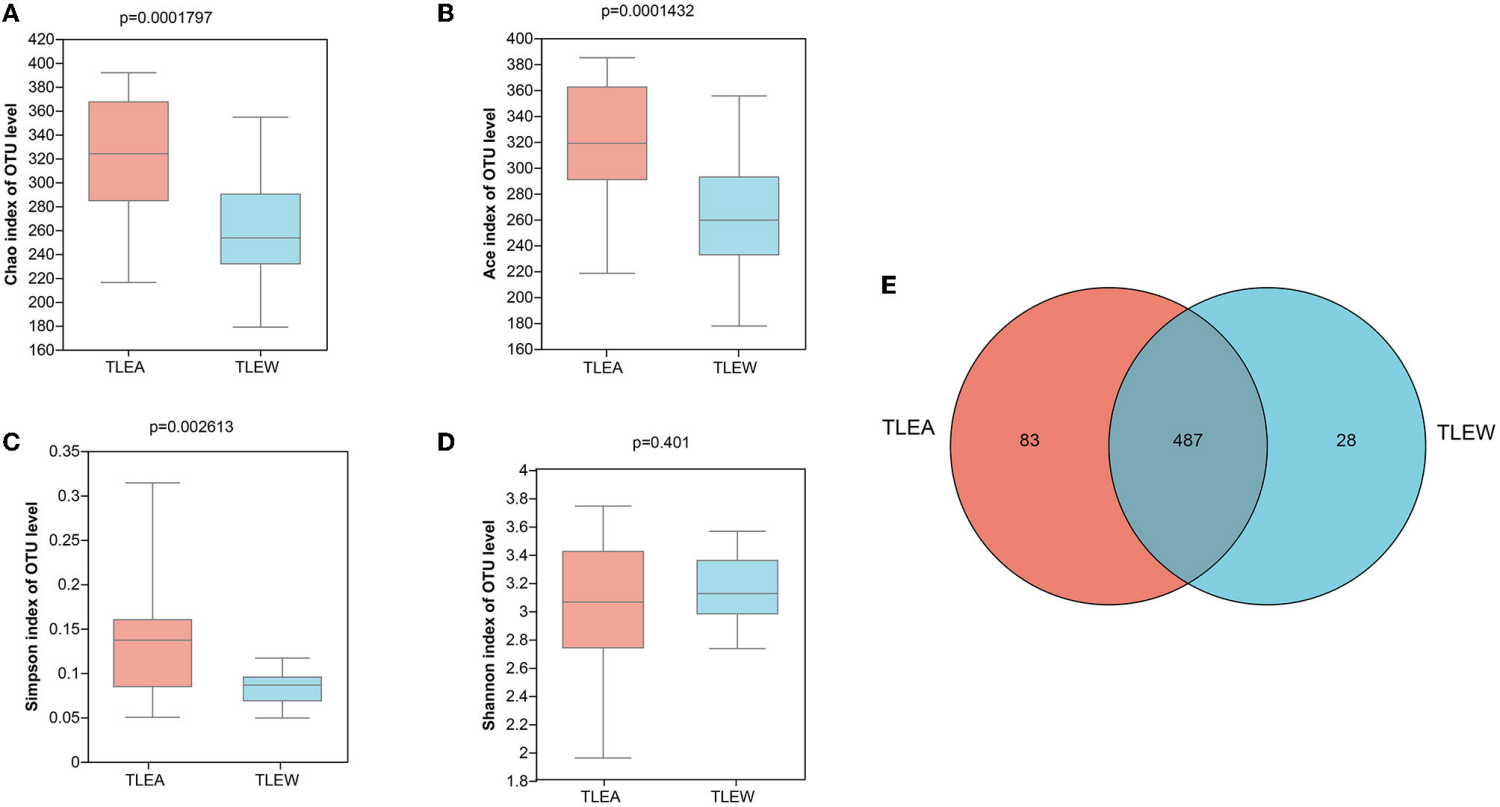
α-diversity of different groups of gut bacteria at the OTU level. **(A–D)** α-diversity of different groups of gut bacteria at the level of operational taxonomic units (OTUs) and index values represent species diversity. **(A)** Variations in Chao **(A)**, Ace **(B)**, Simpson **(C)**, and Shannon **(D)** diversity indices between TLEA and TLEW. **(E)** Comparing the type and number of OTUs. Rare microbial OTUs were eliminated from the Venn diagram, and no subsampling was done. Wilcoxon rank-sum test. TLEA, temporal lobe epilepsy with anxiety disorders; TLEW, temporal lobe epilepsy but without anxiety disorder; OTU, operational taxonomic unit.

The α-diversity of gut bacteria was first assessed. A decrease in microbial richness estimated by Chao and Ace indices was observed in the TLEA group, as compared with the TLEW group (Wilcoxon rank-sum test, *P* < 0.01; Figures 3A, B). The Simpson index showed that bacteria communities in the TLEA group were more abundant than those in the TLEA group (Figure 3C). However, according to the Shannon index, the microfloral diversity in the TLEW group was lower than in the TLEA group, which was, however, not statistically significant (*P* = 0.401; Figure 3D). Venn diagrams were produced to visualize unique and common taxa among the 51 samples (Figure 3E). There were 487 common OTUs in both groups, i.e., the two groups had a high similarity. We found 28 unique OTUs in the TLEA group and 83 unique OTUs in the TLEW group, suggesting a difference in the distribution of the two groups. Our results indicated that bacterial α-diversity was lower in TLEA patients.

### TLEA altered bacterial microbiome structures

**Figure 4:**
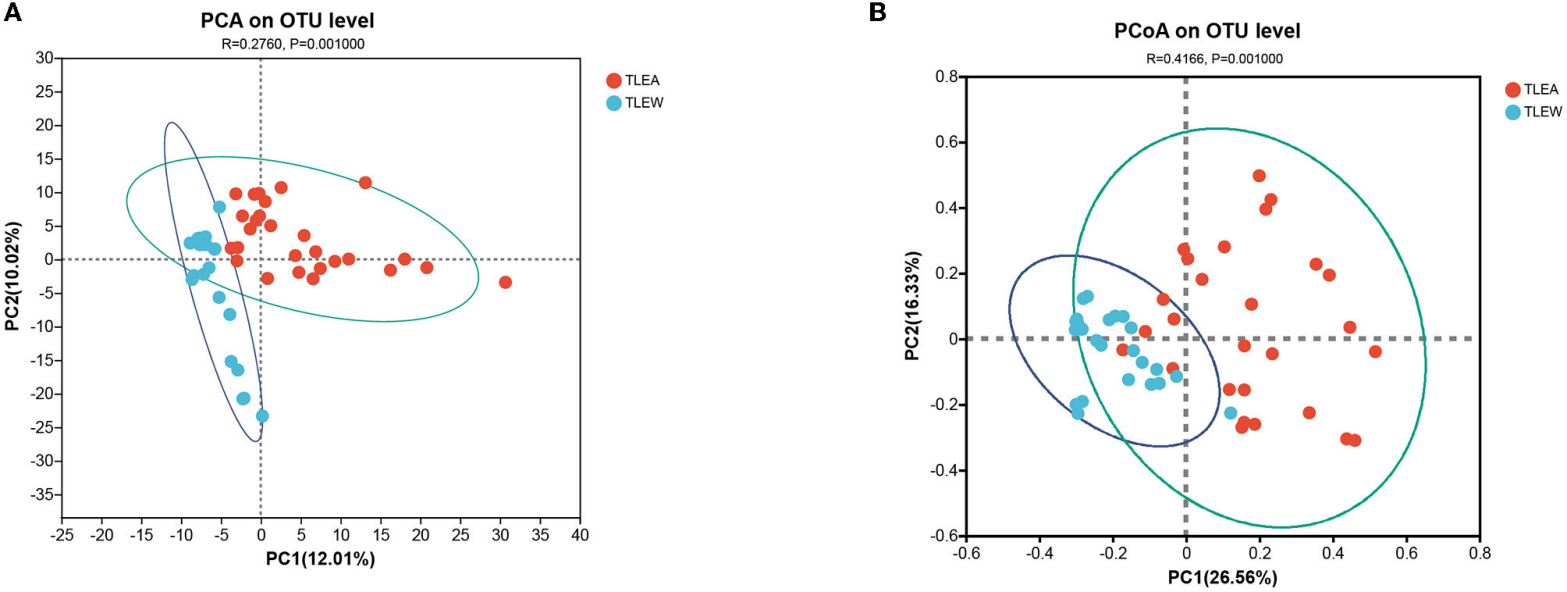
β-diversity of different groups of gut bacteria at the OTU level. Biodiversity of bacterial microbiota. **(A)** PCA of microbiota in TLEA and TLEW patients. **(B)** PCoA of microbiota in TLEA and TLEW patients. TLEA, temporal lobe epilepsy with anxiety disorders; TLEW, temporal lobe epilepsy but without anxiety disorder; OTU, operational taxonomic unit; PCA, principal component analysis; PCoA, principal coordinate analysis.

The bacterial β-diversity has been examined utilizing PCA and PCoA, showing considerable variations across sample clusters as per the OTU level (Figures 4A, B). Samples clustered in the TLEA group, whereas inter-sample distances were larger in the TLEW group. The TLEA group has shown considerably varied bacterial β-diversity in comparison to the TLEW group.

### Bacterial abundance in TLEA

**Figure 5:**
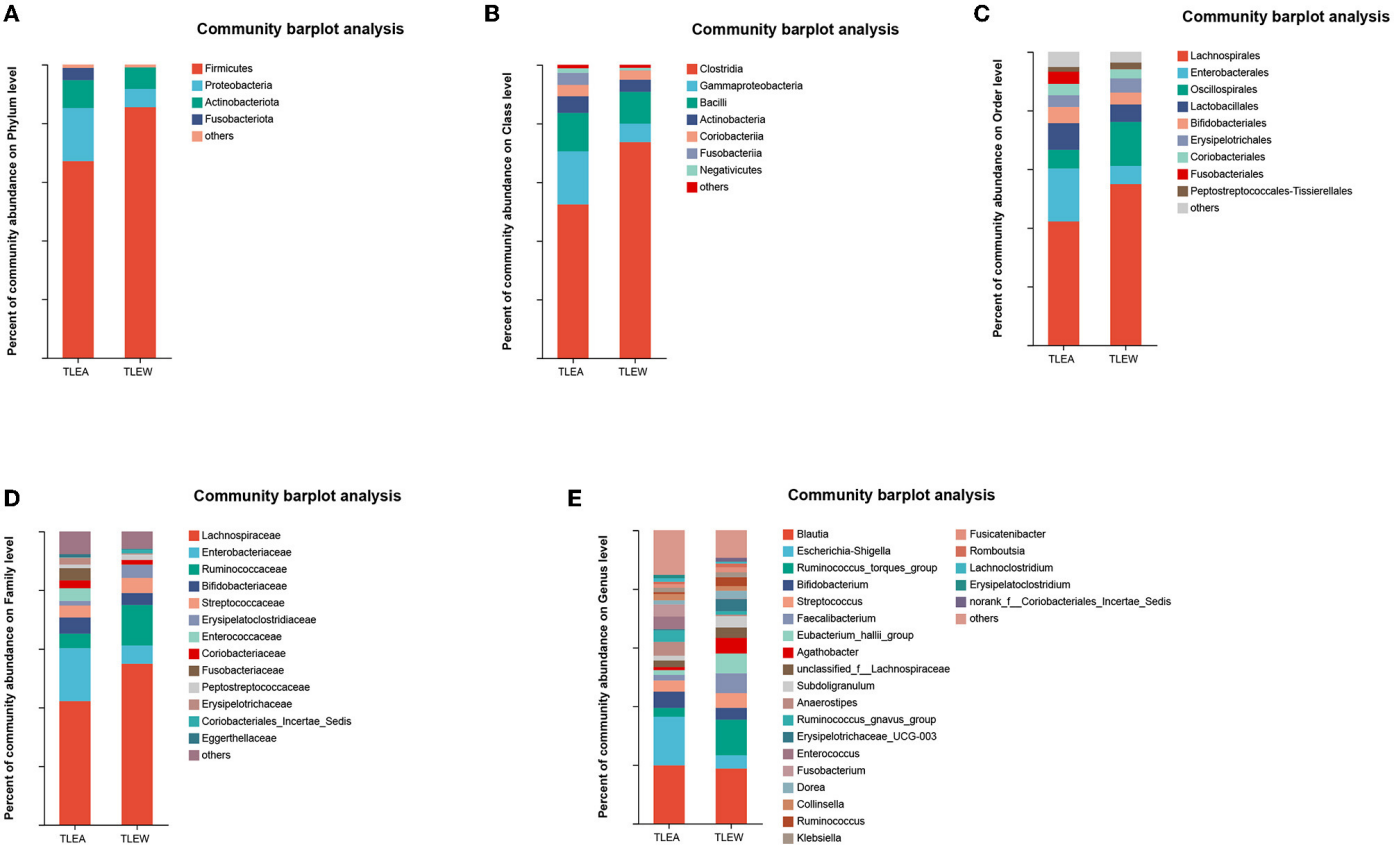
The abundance of gut bacteria in TLEA. The abundance of bacterial communities in TLEA patients at the phylum **(A)**, class **(B)**, order **(C)**, family **(D)**, and genus **(E)** levels. TLEA, temporal lobe epilepsy with anxiety disorders; TLEW, temporal lobe epilepsy but without anxiety disorders.

Operational taxonomic units (OTUs) were compared with the database, and bar graphs were created at the level of phylum, class, order, family, and genus. The results of the distribution of the bacterial community in both groups are shown in Figures 5A–E. In both groups, *Firmicutes, Proteobacteria, Actinobacteriota*, and *Fusobacteriota* were the predominant phyla (other phyla: <0.2%). *Firmicutes* in TLEA samples (67.10%) were significantly less abundant than in TLEW samples, whereas the abundances of *Proteobacteria* (18.08%), as well as *Fusobacteriota* (4.15%), were considerably elevated in TLEA samples (Figure 5A). At the class level, the TLEA group mainly showed *Clostridia* (52.44%), *Gammaproteobacteria* (18.08%), and *Bacilli* (13.09%). *Gammaproteobacteria* were significantly more abundant, and *Clostridia* were less abundant in the TLEA group compared with the TLEW group (Figure 5B). At the order level, the main bacteria in the TLEA group were found as *Lachnospirales* (42.17%), *Enterobacterales* (18.06%), *Oscillospirales* (6.38%), *Lactobacillales* (9.08%), *Bifidobacteriales* (5.55%), *Erysipelotrichales* (4.00%), and *Coriobacteriales* (3.88%). *Lachnospirales* and *Oscillospirales* were significantly less abundant in the TLEA than in the TLEW group, while *Enterobacterales* and *Lactobacillales* abundances were higher in the TLEA than in the TLEW group (Figure 5C). At the family level, the predominant bacteria in the TLEA samples were *Lachnospiraceae* (42.17%), *Enterobacteriaceae* (18.07%), *Ruminococcaceae* (4.92%), and *Bifidobacteriaceae* (5.55%). *Lachnospiraceae* and *Ruminococcaceae* were significantly less abundant in the TLEA group than in the other group. The abundances of *Bifidobacteriaceae* were significantly higher in the TLEA than in the TLEW group (Figure 5D).

At the genus level, *Escherichia*–*Shigella* (12.14%) and *Bifidobacterium* (5.54%) were in considerable abundance in the TLEA group as compared with the TLEW group. *Ruminococcus torques* group (3.00%), *Faecalibacterium* (1.89%), *Eubacterium hallii* group (1.62%), and *Agathobacter* (1.01%) in the TLEA group were significantly less abundant than in the TLEW group (Figure 5E).

### Biomarkers of gut bacteria in TLEA patients

**Figure 6:**
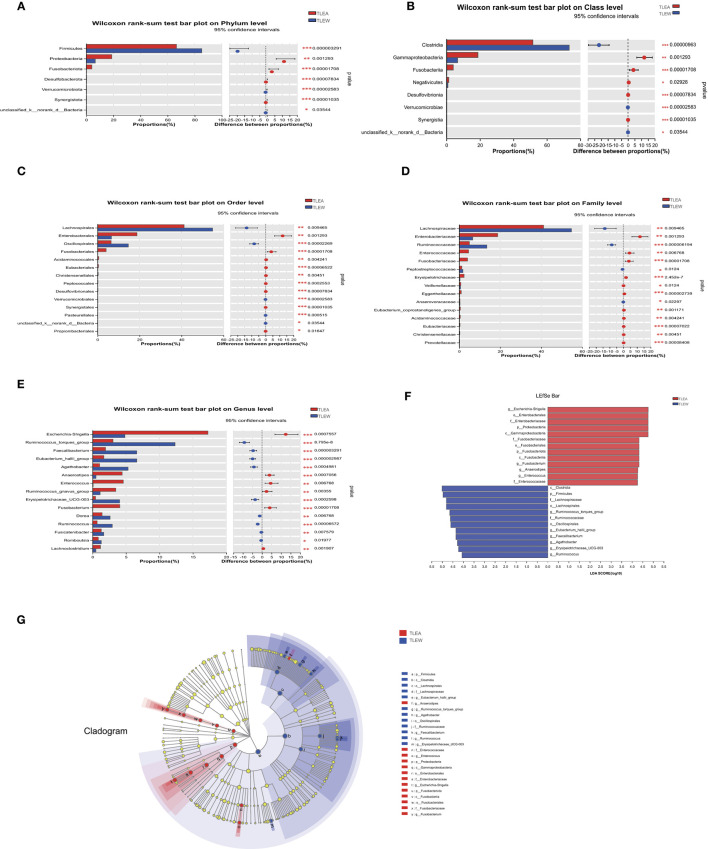
Different levels of bacterial biomarkers in TLEA patients. The abundance of bacterial communities at the phylum **(A)**, class **(B)**, order **(C)**, family **(D)**, and genus **(E)** levels for TLEA samples. **(F)** Macrogenomic biomarkers were analyzed utilizing LEfSe. Significant taxonomic variations in intestinal bacterial communities were identified between the three groups (LDA > 4, non-parametric factor Kruskal–Wallis rank-sum test, *P* < 0.05). **(G)** Branching map of bacterial compartments. Nodes of different colors indicate enriched microbiota and show significant differences between the groups. The size of the circles in the branching map is proportional to fungal abundance. From inside to outside, the circles represent the phylum, order, phylum, and family of fungi, respectively. *P < 0.05; **P < 0.01; and ***P < 0.001. LDA, linear discriminant analysis; LEfSe, LDA effect size; TLEA, temporal lobe epilepsy with anxiety disorders; TLEW, temporal lobe epilepsy but without anxiety disorders.

The LEfSe has been utilized to determine variations in metagenomic biomarkers across the two groups. As shown in Figure 6, the biomarkers of TLEA were Proteobacteria and *Fusobacteriota* at the phylum level (Figures 6A, F, G); *Gammaproteobacteria* and *Fusobacteriia* at class level (Figures 6B, F, G); *Enterobacterales* and *Fusobacteriales* at the order level (Figures 6C, F, G); *Enterobacteriaceae, Fusobacteriaceae Enterococcaceae* at the family level (Figures 6D, F, G); *Escherichia–Shigella, Fusobacterium, Anaerostipe* and *Enterococcus* at the genus level (Figures 6E–G). In contrast, *Clostridia* at class level (Figures 6B, F, G); *Lachnospirales* and *Oscillospirales* at the order level (Figures 6C, F, G); *Firmicutes* and *Lachnospiraceae Ruminococcaceae* at the family level (Figures 6D, F, G); *Eubacterium hallii* group, *Faecalibacterium, Agathobacter, Erysipelotrichaceae* UCG-003, and *Ruminococcus* torques group at the genus level (Figures 6E–G), showed higher abundances in the TLEW group.

Furthermore, we found that compared with male patients, female patients with TLEA had lower bacterial abundances in *unclassified*_c__*Clostridia* (order), *Eubacterium*_*coprostanoligenes*_*group* (family) *norank*_f__*Eubacterium*_*coprostanoligenes*_*group* (genus), *Lachnospiraceae_FCS020*_group (genus), and *Marvinbryantia* (genus) but higher in *Bacteroidaceae* (family), *Bacteroides* (genus), *Bacteroides*_*thetaiotaomicron* (species), *uncultured*_*bacterium*_g__*Sellimonas* (species), *Bacteroides*_*uniformis* (species), *Bacteroides*_*dorei* (species), and *Bacteroides*_*caccae* (species) (Supplementary Figures 1A–D). Moreover, the abundance of TLEA bacteria varied with the different treatment regimens, including *Micrococcales* (order), *Micrococcaceae* (family), *Morganellaceae* (family), *Morganella* (genus), *uncultured*_*organism*_g__norank_f__*Ruminococcaceae* (species), *Streptococcus*_*agalactiae* (species), *Streptococcus*_*sobrinus* (species), and *Morganella*_*morganii* (species) (Supplementary Figures 2A–D).

### Diversity of fungal microbiota in TLEA patients

**Figure 7:**
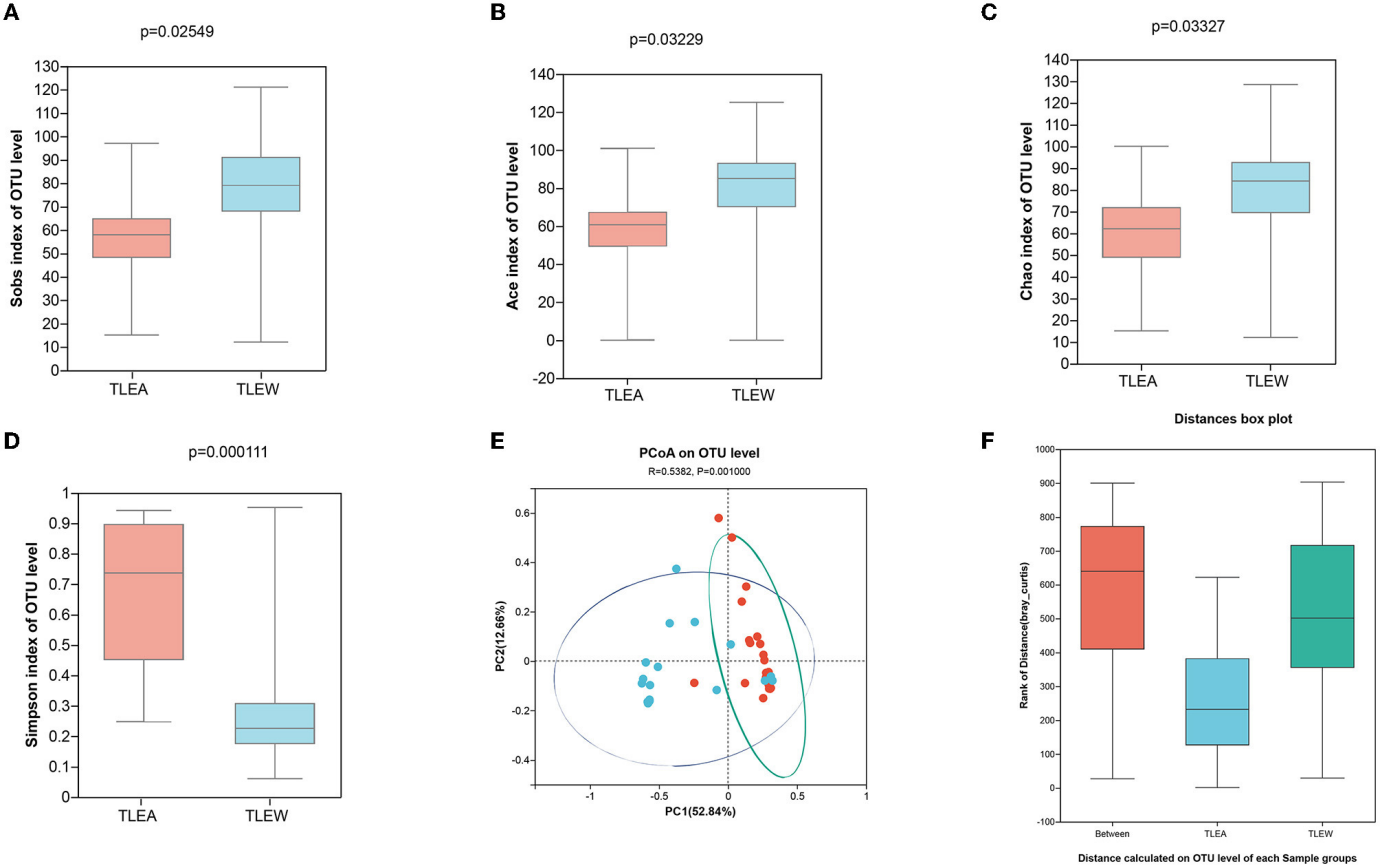
Diversity of gut fungi in TLEA patients. Sobs **(A)**, Ace **(B)**, Chao **(C)**, and Simpson **(D)** differences in OTU levels of fungal flora in patients with TLEA and TLEW groups. Values represent the diversity of species. **(E)** Analysis of fungal β-diversity utilizing principal component analysis (PCA). There was no significant clustering among the groups of samples. **(F)** Analysis of similarity between fungi (ANOSIM/Adonis). The vertical coordinate indicates the distance rank calculated at the level of operational taxonomic units (OTUs) for each group. TLEA, temporal lobe epilepsy with anxiety disorders; TLEW, temporal lobe epilepsy but without anxiety disorder; OTU, operational taxonomic unit; ANOSIM, analysis of similarities; TLEA, temporal lobe epilepsy with anxiety disorders; TLEW, temporal lobe epilepsy but without anxiety disorders.

The Sobs, Ace, and Chao indices on an OTU level have shown that fungal α-diversity was reduced for the TLEA group, as compared with the TLEW group (*P* < 0.05), whereas the Simpson index of fungal α-diversity displayed a non-significant increase for the TLEA than in the TLEW group (Figures 7A–D). Based on the β-diversity index, the PoCA showed significant differences between the fungal microbiota of the TLEA and TLEW groups (Figure 7E). Similarity analysis revealed that the distances derived at the OTU level for each sample varied considerably (ANOSIM/Adonis, Figure 7F; *P* < 0.05).

### Composition and structure of the fungal microbiota in TLEA

**Figure 8:**
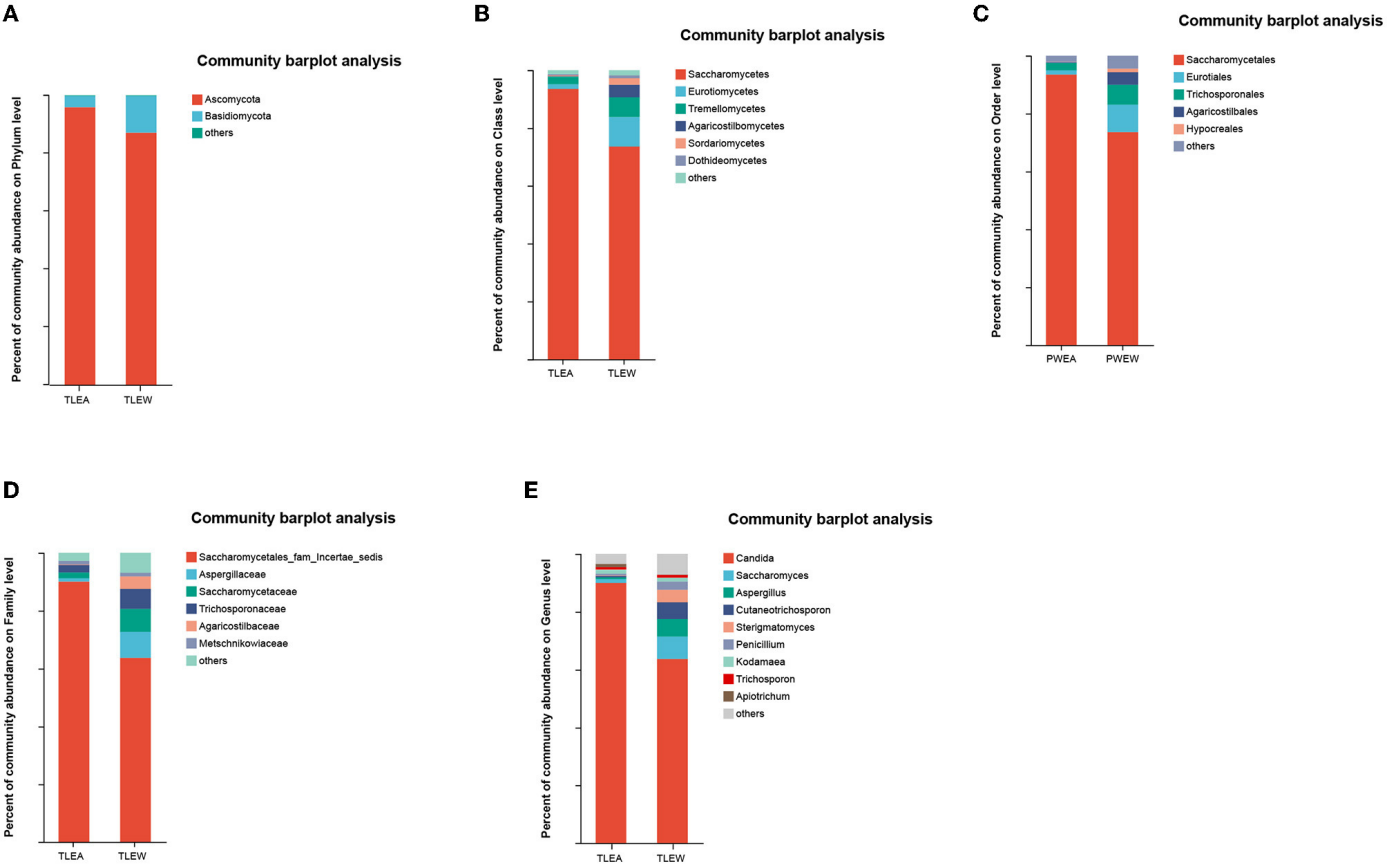
The abundance of fungal communities in TLEA patients at the phylum **(A)**, class **(B)**, order **(C)**, family **(D)**, and genus **(E)** levels. TLEA, temporal lobe epilepsy with anxiety disorders; TLEW, temporal lobe epilepsy but without anxiety disorders.

*Ascomycota*, as well as *Basidiomycota*, were the most prevalent phyla found for the gut fungal microbiota. At the phylum level, the proportion of *Ascomycota* (95.51%) was higher in the TLEA than in the TLEW group, while that of *Basidiomycota* (3.87%) was significantly lower in the TLEA group (Figure 8A). At the class level, lowered levels of *Eurotiomycetes* (1.70%) and *Tremellomycetes* (2.54%), as well as elevated levels of *Saccharomycetes* (93.50%), have been found for the TLEA group (Figure 8B). At the order level, the TLEA group showed lower abundances of *Eurotiales* and *Trichosporonales* and higher abundances of Saccharomycetales (Figure 8C). At the family level, the abundances of *Aspergillaceae, Saccharomycetaceae, Trichosporonaceae, Agaricostilbaceae*, and *Metschnikowiaceae* were lower, and those of *Saccharomycetales fam*. *incertae sedis* were higher in the TLEA group (Figure 8D). At the genus level, *Saccharomyces, Aspergillus, Cutaneotrichosporon, Sterigmatomyces*, and *Penicillium* were less abundant in the TLEA group (Figure 8E).

**Figure 9:**
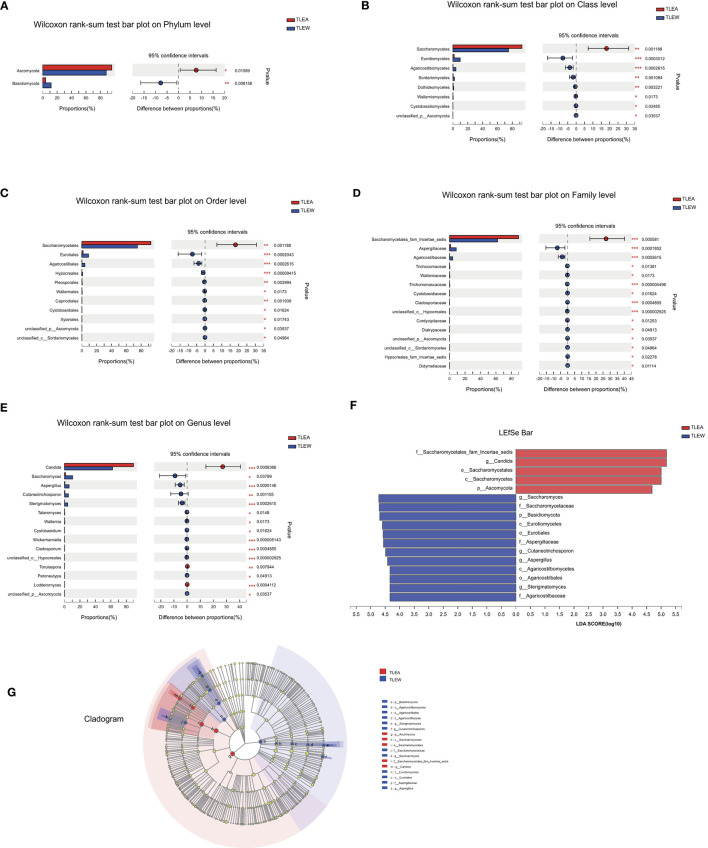
Different levels of fungal biomarkers in TLEA patients. The abundance of fungal communities at the phylum **(A)**, class **(B)**, order **(C)**, family **(D)**, and genus **(E)** levels for TLEA samples. **(F, G)** LEfSe bar plot of fungal communities. Linear discriminant analysis (LDA) has been performed to assess the influence of each component's abundance on differential effects. *P < 0.05; **P < 0.01; and ***P < 0.001. LDA, Linear discriminant analysis; LEfSe, LDA effect size; TLEA, temporal lobe epilepsy with anxiety disorders; TLEW, temporal lobe epilepsy but without anxiety disorders.

The Wilcoxon rank-sum tests also indicated considerable variations in fungal microbiota abundance among phylum, class, order, family, and genus, including *Ascomycota* (phylum), *Saccharomycetes* (class), *Saccharomycetales* (order), *Saccharomycetales* fam. *incertae sedis* (family), and *Candida* (genus) (Figures 9A–E).

Furthermore, we found that, compared with male patients with TLEA, female patients had lower bacterial abundances in *Malasseziomycetes* (class), *Agaricomycetes* (class), *Malasseziales* (order), *Filobasidiales* (order), *Mala*sseziaceae (family), and *Malassezia* (genus) (Supplementary Figures 3A–D). Moreover, the abundances of TLEA bacteria varied with different treatment regimens, including *Sordariales* (order), *Chaetomiaceae* (family), *Morganellaceae* (family), *Pichia* (genus), *Choanephora* (genus), *Candida*_*orthopsilosis* (species), *Penicillium*_*allii* (species), *Cladosporium*_*delicatulum* (species), and *Choanephora*_*cucurbitarum* (species) (Supplementary Figures 4A–D).

LEfSe plots, along with cladograms, illustrated the fungal microbiota to be the most pronounced variations in terms of relative abundance (Figures 9F, G). In the TLEA group, *Saccharomycetales* fam. *incertae sedis* (family), Saccharomycetales (order), *Saccharomycetes* (class), and *Ascomycota* (phylum) were significantly more abundant than in the TLEW group (LDA = 4.0).

### Predicted microbial functions altered in TLEA patients

**Figure 10:**
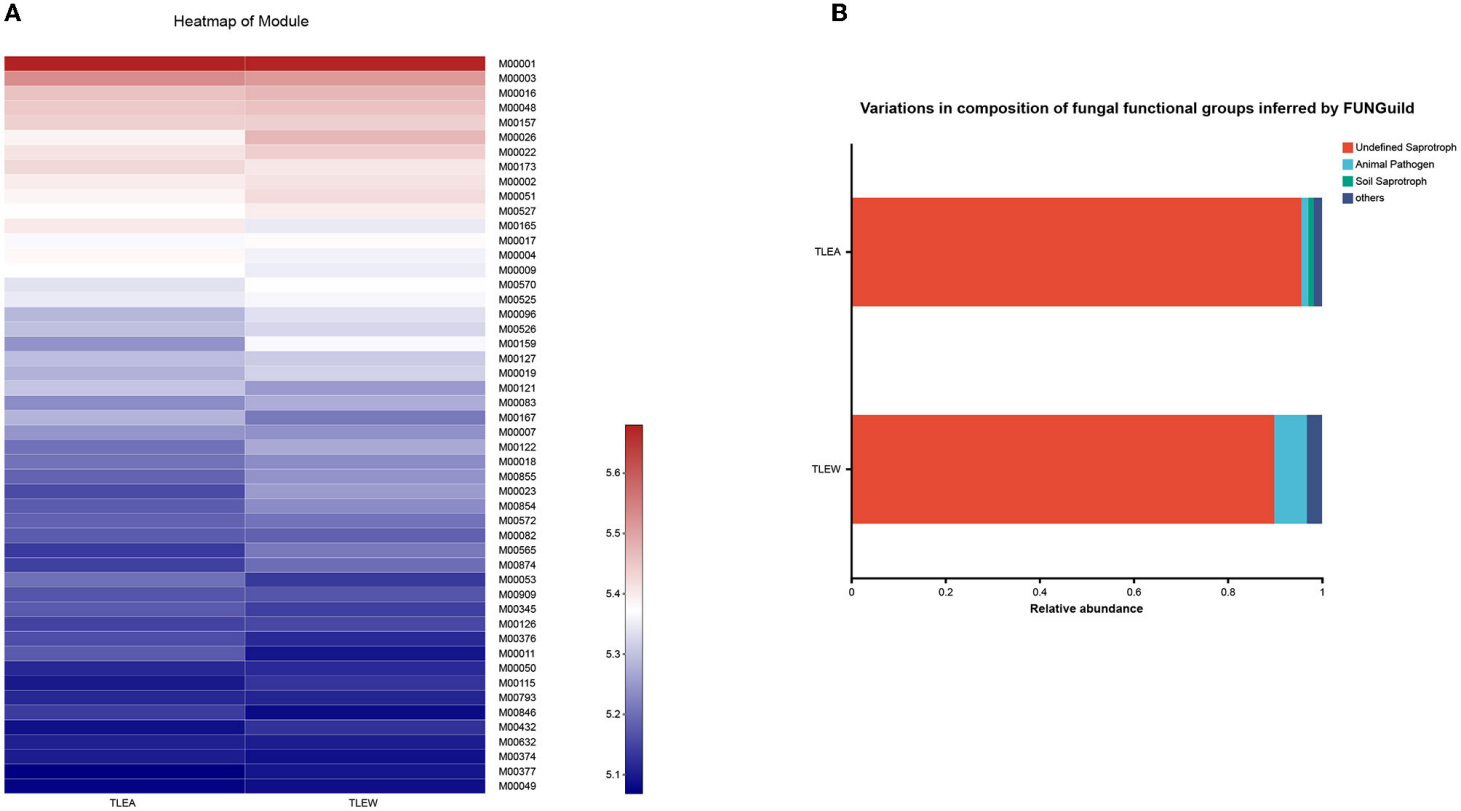
Predicted microbial functions altered in TLEA patients. **(A)** A heatmap of the module based on PICRUSt 1. Differences among TLEA and TLEW. **(B)** Variations in the composition of fungal functional groups inferred by FUNGuild. Differences in fungal functional groups among TLEA and TLEW patients. TLEA, temporal lobe epilepsy with anxiety disorders; TLEW, temporal lobe epilepsy but without anxiety disorders.

TLEA gut bacteria have been characterized utilizing PICRUSt's predictions of functional compositions from 16S rRNA sequencing data. Several KEGG (module level) categories such as uridine monophosphate biosynthesis (M00051), glycogen biosynthesis (M00854), glycogen degradation (M00855), C5 isoprenoid biosynthesis, non-mevalonate pathway (M0096), trehalose biosynthesis (M00565), cobalamin biosynthesis (M00122), tryptophan biosynthesis (M0023), and histidine biosynthesis (M00026) were lower in the TLEA group (Figure 10A). Ascorbate degradation (M00550), reductive pentose phosphate cycle (Calvin cycle) (M00165), reductive pentose phosphate cycle (M00167), KDO2-lipid A biosynthesis, Raetz pathway (M00866), citrate cycle, second carbon oxidation (M0011), KDO2-lipid A biosynthesis, LpxL-LpxM type (M00060), menaquinone biosynthesis (M00116), formaldehyde assimilation, xylulose monophosphate pathway (M00344), pyrimidine deoxyribonucleotide biosynthesis (M0053), and heme biosynthesis (M00121) were enriched in the TLEA group.

The FunGuild database was used to assign fungal OTUs to specific functional groups, and identified guilds are shown in Figure 10B. Undefined saprotroph accounted for ~90% of all detected fungal OTUs. TLEA had a higher relative abundance of saprotrophs of uncertain taxonomic classification as compared with TLEW, whereas TLEW possessed a higher relative abundance of animal pathogens.

Taken together, these results point to the possibility that changes in the composition of the host's microbial community may disrupt the host's physiological processes.

### Relationship between microbial community structures and TLEA AIFs

**Figure 11:**
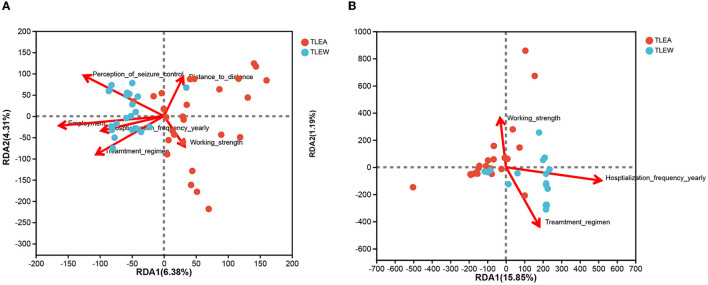
RDA of anxiety impact factors (arrows). Bacterial and fungal flora are shown in **(A, B)**, respectively. The values of axis 1 and axis 2 are the percentages explained by the corresponding factor. RDA, redundancy analysis; TLEA, temporal lobe epilepsy with anxiety disorders; TLEW, temporal lobe epilepsy but without anxiety disorders.

Anxiety impact factors (AIFs) are involved in the progression of TLEA and may affect the structure of TLEA intestinal bacterial and fungal microbial communities to varying degrees (Table 1 and Supplementary Table 1). Therefore, an attempt is made to investigate whether AIF affects microbial community structures. AIF with statistically significant variations was included in the RDA. RDA of bacteria was conducted using six factors, i.e., employment, working strength, distance to distance, annual hospitalization frequency, treatment regimen, and perception of seizure control (Figure 11A). Fungal RDA identified three risk factors, namely, working strength, annual hospitalization frequency, and treatment regimen (Figure 11B). Employment (*P* = 0.009) and perception of seizure control (*P* = 0.019) significantly affected TLEA bacterial community structure, whereas annual hospitalization frequency (*P* = 0.014) significantly affected TLEA fungal community structures.

## Discussion

Although research has linked altered gut microbiota to disease etiology in epileptic patients, the link between gut microbiology and TLEA remains obscure. Intestinal microbes and fungi were compared between TLEA and TLEW patients for the first time in this research. The results from this study illustrate significant variations in gut flora composition and functional pathways between TLEA and TLEW patients.

The gut microbiota can influence the central as well as enteric nervous systems through a variety of mechanisms, such as the production and expression of neurotransmitters and neurotrophic factors, regulation of intestinal sensory afferents, metabolite production, mucosal immune regulation, and maintenance of the integrity of the intestinal barrier and tight junctions (Młynarska et al., 2022). TLEA patients endure enormous stress. The stress decreases the specific proteins of intestinal epithelial tight junction, such as Recombinant Claudin 1 (Da Silva et al., 2014), and disrupts the integrity of the intestinal epithelium, modifying the intestinal motility, secretions, and mucin production (Wong et al., 2016; Rutsch et al., 2020). On the other hand, we (Wei et al., 2020) and previous studies (Keita and Söderholm, 2018; Serek and Oleksy-Wawrzyniak, 2021) also found that disturbance of intestinal flora would increase the permeability of intestinal mucosal membranes, leading to the brain inflammation. One of the most relevant is the translocation of outer membrane vesicles and allergens produced by gram-negative bacteria into the bloodstream, which will trigger brain inflammation signaling (Wong et al., 2016; Rutsch et al., 2020; Wei et al., 2020). Thus, we conclude that unbalanced fungal diversity and abundance may contribute to the intestinal inflammatory process or increase the development of TLEA.

Considerable alterations in gut bacterial composition were also identified in TLEA patients. This was corroborated by α-diversity analysis that revealed a reduced gut bacteria diversity in the TLEA group. Moreover, the β-diversity analyses indicated that the TLEA group had clusters of gut bacteria that differed from those of the TLEW group, which clustered together. At the phylum level, there was a significant drop in Firmicutes and an increase in *Proteobacteria* and *Fusobacteriota* in TLEA patients. Anxiety disorder patients have lower microbial richness than those without anxiety (Jiang et al., 2018). In a cross-sectional study, Jiang et al. reported a significant increase in the phylum *Firmicutes* (especially, *Lachnospira* and the anti-inflammatory symbiotic *Faecalibacterium*) and a significant decrease in *Fusobacteria* (Jiang et al., 2018). This is consistent with the finding for patients with TLEA. In particular, *Fusobacteriaceae*, an invasive and pro-inflammatory pathogen, increased from the family level to the genus level of intestinal microorganisms in TLEA. Previous microbiome investigations in individuals with epilepsy and Alzheimer's disease have also shown similar alterations and reductions in bacterial diversity (Safak et al., 2020; Lim et al., 2022).

At the family level, *Lachnospiraceae* and *Ruminococcaceae* were less abundant in the TLEA group. These symbiotic bacteria are associated with intestinal health. Lachnospiraceae are involved in human energy supply and immunomodulatory functions (Arpaia et al., 2013; Pascale et al., 2018) while maintaining health by producing short-chain fatty acids, converting primary bile acids into secondary bile acids, and averting colonization by intestinal pathogens (Byndloss et al., 2017; Lan et al., 2022). It has been hypothesized that the gut microbiota of TLEA patients may be more vulnerable than that of TLEW patients when concomitant anxiety occurs, which may be due to their prolonged medication use and lack of timely adjustment of medication regimens, among other reasons. However, this conclusion is limited by the sample size of this study and requires confirmation. In addition, *Ruminococcus* proliferate during symptomatic episodes in patients with Crohn's disease and produce inflammatory polysaccharides (Yan et al., 2022). A reduction of *Ruminococcus gnavus* was found in both anxious (Jiang et al., 2018) and TLEA patients. It is hypothesized that lower levels of *Lachnospiraceae* and *Ruminococcaceae* may contribute to the disruption of intestinal functioning and increased intestinal mucosal inflammation in TLEA patients.

At the genus level, we and another study of 36 anxiety disorder patients (Chen et al., 2019) demonstrated a greater enrichment in *Escherichia*–*Shigella* in patients with anxiety. It is noteworthy that Chen also observed a positive association between *Escherichia*–*Shigella* and the severity of anxiety. Other groups such as *Enterobacteriaceae* (Chen et al., 2019), *Enterobacteriales* (Chen et al., 2019), and *Proteobacteria* (Dong et al., 2021) not only increased in anxiety disorder patients but also TLEA patients, highlighting the relationship between the presence of pathogens in the gut and anxiety again. Nevertheless, the observation is inconsistent with data from children with autism and epilepsy (Dan et al., 2020; Safak et al., 2020). *Escherichia*-*Shigella* include bacteria with pro-inflammatory activity (Reinoso Webb et al., 2016), which causes intestinal inflammation by bacterial structural components (i.e., microbial-associated molecular patterns) such as lipopolysaccharides and bacterial metabolism (Ceccarani et al., 2020). With the exception of *Escherichia*-*Shigella*, TLEA patients showed higher abundances of Gram-negative bacteria such as *Enterobacteriaceae*, compared with TLEW patients. *Lipopolysaccharides* are a major cell wall component in Gram-negative bacteria that assists in binding to Toll-like receptor 4 (TLR4) while activating MyD88-dependent signaling pathways in the lamina propria, leading to secretion of pro-inflammatory mediators, which elicit and sustain local inflammation and promote seizures (Ceccarani et al., 2021). In addition, activation of the TLR4 signaling pathway promotes neuroinflammation (Paudel et al., 2020). GABA type A receptor α1 binding and negative regulation of TLR4 leads to epilepsy–migraine comorbidity, and TLR4 is a key intermediate in epilepsy–migraine comorbidity (Lin et al., 2022). These studies provide further evidence that epilepsy and anxiety synergistically contribute to gut dysfunction.

In short, *Faecalibacterium, Fusobacteria, Lachnospiraceae, Ruminococcaceae*, and *Escherichia*–*Shigella* were not only different in anxious and healthy people but also different in TLEW and TLEA. We infer that the bacteria mentioned are also different in TLEA and healthy individuals.

Interestingly, we also found a reduced abundance of probiotics such as the *Ruminococcus torques* group, *Faecalibacterium, Eubacterium hallii* group, and *Agathobacter* in TLEA patients. These probiotics are reduced in inflammation-associated microbiota (Bai et al., 2022; Bonnechère et al., 2022). This suggests that the TLEA intestinal microenvironment is detrimental to the growth of bacterial strains that exert anti-inflammatory effects, which indirectly promotes the development of anxiety disorders.

The biodiversity of fungal microbiota in TLEA patients is low. The fungal microbiota is often considered a relatively small part of the gut microbiota, representing ~0.1%. As fungal taxa are rare in existing genomic databases, the role of the fungal microbiota in the human gut remains a mystery (Wang et al., 2022). An aberrant growth of several taxa in the fungal microbiome that is linked to opportunistic infections and inflammation has been observed. Consistent with the altered bacterial diversity reported in the TLEA group, we found that fungal α-diversity was lower in the TLEA group. However, significant biodiversity indices are insufficient in TLEA. The composition differences of the fungal flora in the TLEA group were fewer than that of the bacterial flora, and the individual differences were greater.

Although there was little change in fungal abundance in TLEA compared with TLEW patients, a trend toward decreased fungal diversity occurred in the TLEA group. Thus, there may be an association between bacteria and fungi in the development of epileptic disorders. The change in fungal abundances was not significant compared with that of bacteria, probably because of the insufficient sample size, the small proportion of fungi, and the lack of comprehensive fungal databases. More research with larger sample sizes and sophisticated assay methods is required to identify the involvement of fungi and the link between bacteria and fungi in the onset and progression of TLEA.

We found an increased proportion of *Ascomycota* and *Candida albicans* in the TLEA group. Interestingly, Zheng et al. have also reported an elevated proportion of *Candida albicans* in fecal fungal microbiota in patients with intestinal diseases (Zeng et al., 2022). *Candida albicans* is thought to be an inducer of T helper 17 cells, which are involved in the immunity of the intestinal mucosal barrier (Zeng et al., 2022). Under pathological conditions, such as during inflammatory bowel disease, T helper 17 cells secrete pro-inflammatory cytokines that exacerbate intestinal inflammation (Zeng et al., 2022), suggesting that *Candida albicans* may promote anxiety in epilepsy.

The composition of the intestinal bacterial and fungal flora is highly influenced by gender, medication, geography, and food (Chowdhury and Fong, 2020; De and Dutta, 2022). Unfortunately, not all previous studies have examined the effects of medications on gut microbiota profiles. Only one study of eight female participants exclusively diagnosed with anxiety was performed in the Caucasian population (Mason et al., 2020). In Mason's study, neither α-diversity nor β-diversity was significantly associated with anxiety, contrary to us and other previous studies where anxiety was associated with a lower fecal bacterial α-diversity. Similarly, the abundance of *Bacteroides* was also inconsistent with previous studies.

The Han Chinese epilepsy population in tropical China recruited for this study had a regular diet and lifestyle and exhibited similar dietary habits and lifestyles. The biases of diet and geographic and ethnic factors are likely minimal. Furthermore, the effect of gender and treatment regimen on intestinal microbial composition between the groups was observed in the study. We investigated the effects of the treatment regimen on the profiles of intestinal microbiota. There was an increase in the abundance of *Morganella* (family), *Morganella* (genus), and *Morganella morganii* (species) in TLEA patients treated with polytherapy vs. monotherapy. Considered to be a significant opportunistic pathogen, *Morganella morganii* can cause various infections, such as septicemia, abscess, chorioamnionitis, cellulitis, and purple urine bag syndrome (Liu et al., 2016). Accumulated data have demonstrated that the virulence of evolution makes *Morganella morganii* a major pathogen (Liu et al., 2016). From these results, we can conclude that long-term polytherapy in epileptic patients will lead to the development of resistance to harmful bacteria and the disorder of intestinal flora. An increase in unusual opportunistic pathogens is a serious challenge in addressing clinical infections.

We also found that *Bacteroides* increased significantly in women. Specifically, the abundance of *Bacteroides* is responsible for the deconjugation of conjugated bile acids synthesized in the liver (Siddiqui et al., 2022). *Bacteroides vulgatus* was significantly higher in women with polycystic ovary syndrome compared with controls (Qi et al., 2019; Siddiqui et al., 2022). We inferred that the TLEA female patients had disordered intestinal flora structures and increased *Bacteroides* compensatively. Due to the bias in the limited sample size, the value is limited, and these conclusions should be analyzed with caution. Overall, TLEA has similarities with the microbiome found in anxious patients, suggesting that microbiome modulation may be a preventative and therapeutic tool for TLEA.

At the functional level, a unique microbial metabolic pathway profile was present in TLEA's gut, such as ascorbic acid synthesis. Vitamin C(ascorbic acid) is a well-known antioxidant that is said to be involved in treating anxiety in humans (Oliveira et al., 2015; Pratiwi et al., 2019). Vitamin C supplementation at 3,000 mg daily lowered subjective stress against acute psychological stressors (Brody et al., 2002), and a relief effect on anxious mood was observed after vitamin C administration in healthy individuals (Oliveira et al., 2015; Moritz et al., 2017). Further studies will be needed to monitor changes in neurotransmitters, neurohormones, and neurotrophins in TLEA patients to understand the underlying mechanisms by which vitamin C affects TLEA brain function. Uridine, a precursor to cytidine diphosphate-choline, is involved in phospholipid synthesis, being evaluated as a potential medication for bipolar depression (Agarwal et al., 2011). Notably, nearly half of the patients with bipolar disorder have anxiety disorders during their lifetime (Qs et al., 2021). Our study found reduced uridine monophosphate biosynthesis in the intestinal microbiota of TLEA patients. This suggests that uridine supplementation might also modulate the imbalance of intestinal flora of TLEA. Further investigations are required to clarify the effects of uridine on anxiety disorder.

As a potent immunoreactive factor, Lipid A can be recognized by animal cells and triggers defense-related responses, causing gram-negative sepsis and endotoxic shock (Opiyo et al., 2010). We observed the KDO2-lipid A biosynthesis enriched in the TLEA group, indicating an increased risk of intestinal inflammation in TLEA patients.

In addition, we also found that several metabolic pathways in TLEA patients' gut microbiota are directly or indirectly related to anxiety, including tryptophan biosynthesis (M0023) (Songtachalert et al., 2018; Evrensel et al., 2020), reductive pentose phosphate cycle (Calvin cycle) (Peng et al., 2022), Raetz pathway (Nguyen et al., 2020), and menaquinone biosynthesis (Johnston and Bulloch, 2020). Together, these findings enrich TLEA's brain–gut axis studies.

Moreover, work-related factors such as employment and working strength increased anxiety in people with epilepsy. This is because of the psychological burden that can be caused by long periods of unemployment and working strength. Patients frequently develop guilt and fear of unemployment due to the disease. To better interpret the pathophysiology of TLEA and design individualized methods to change the gut microecology in TLEA patients, it is important to have a better understanding of the regulatory activities of bacteria in the gut.

## Limitations

This study has some limitations. First, owing to the short sample size, a subgroup analysis of TLEA anxiety levels was not conducted. Possible mechanisms for the effect of gut flora on epilepsy remain to be elucidated, which requires further characterization of the mechanisms of gut microbiota involvement in epileptogenesis using more extensive sample-size studies based on macrogenomics, proteomics, and metabolomics. Second, data specific to the gut inflammation and permeabilities of the patients with TLEA are also essential for future research. Gut inflammation and permeabilities of TLEA will need to be compared with TLEW, including whether specific taxa associated with the gut inflammation and permeabilities can influence the development of TLEA. In addition, the microbiota at the species level was not investigated due to the unknown functions of many species. Current techniques and databases are not sufficiently comprehensive to allow a complete understanding of the functions of gut microbes. Moreover, the role of selecting specific gut microbiota in the development and progression of TLEA requires further investigation. Probiotics have been utilized to regulate the composition of the gut microbiota in patients with a variety of diseases in recent years. Transplanting probiotics or enterobacteria other than specific bacteria or fungi into the gut of animal models of mice would be interesting to see if the disease progression is successfully slowed, and if new therapeutic approaches for TLEA are offered.

Finally, the lack of control groups in the current study (i.e., groups of healthy controls and patients with anxiety disorders but without epilepsy) represents the main limitation. Therefore, future studies should be planned to include the use of a group of healthy controls, for instance, patients' relatives could reduce some bias related to environmental stressors, lifestyle, and diet, which are likely to contribute to gut microbiota dysbiosis. Many of the physical and mental health conditions are correlated with the gut microbiota. Recruiting individuals with anxiety disorder without epilepsy will be crucial in the next phase of the investigation, which could help identify the influence of this psychiatric disorder on gut microbiota in this specific geographical location.

## Data availability statement

The datasets presented in this study can be found in online repositories. The names of the repository/repositories and accession number(s) can be found in the article/Supplementary material.

## Ethics statement

The studies involving human participants were reviewed and approved by the Clinical Trials Ethics Committee Central People's Hospital of Zhanjiang (approval number: PJI117-2022-027-013) approved the study protocols. The patients/participants provided their written informed consent to participate in this study.

## Author contributions

SW, JW, and YC conceived and designed the study. RZ, YM, DZ, and LH carried out the statistical analysis and interpretation of data. All authors contributed toward drafting and revising the manuscript and agree to be accountable for all aspects of the manuscript.

## Supplementary material

Supplementary Figure 1 TLEA subgroup analysis. The abundance of bacterial community at order **(A)**, family **(B)**, genus **(C)**, and species **(D)** levels in different gender. TLEA, temporal lobe epilepsy with anxiety disorders.

Supplementary Figure 2 TLEA subgroup analysis. The abundance of bacterial community order **(A)**, family **(B)**, genus **(C)**, and species **(D)** levels in the different treatment regimens. TLEA, temporal lobe epilepsy with anxiety disorders.

Supplementary Figure 3 TLEA subgroup analysis: the abundance of gender fungal community at class **(A)**, order **(B)**, family **(C)**, and genus **(D)** levels. TLEA, temporal lobe epilepsy with anxiety disorders.

Supplementary Figure 4 TLEA subgroup analysis: the abundance of treatment regimen fungal community at order **(A)**, family **(B)**, genus **(C)**, and species **(D)** levels. TLEA, temporal lobe epilepsy with anxiety disorders.

Supplementary Table 1 Redundancy analysis (RDA) of anxiety impact factors.

Supplementary Table 2 Characteristics of 45 temporal lobe epilepsy patients^*^.

Supplementary Table 3 Redundancy analysis (RDA) of anxiety impact factors.
