# Supplementary figures and images for: Altered gut microbiota in temporal lobe epilepsy with anxiety disorders
Source: Front Microbiol. 2023 May 22;14:1165787. doi: 10.3389/fmicb.2023.1165787 (PMC10239838; doi:10.3389/fmicb.2023.1165787)

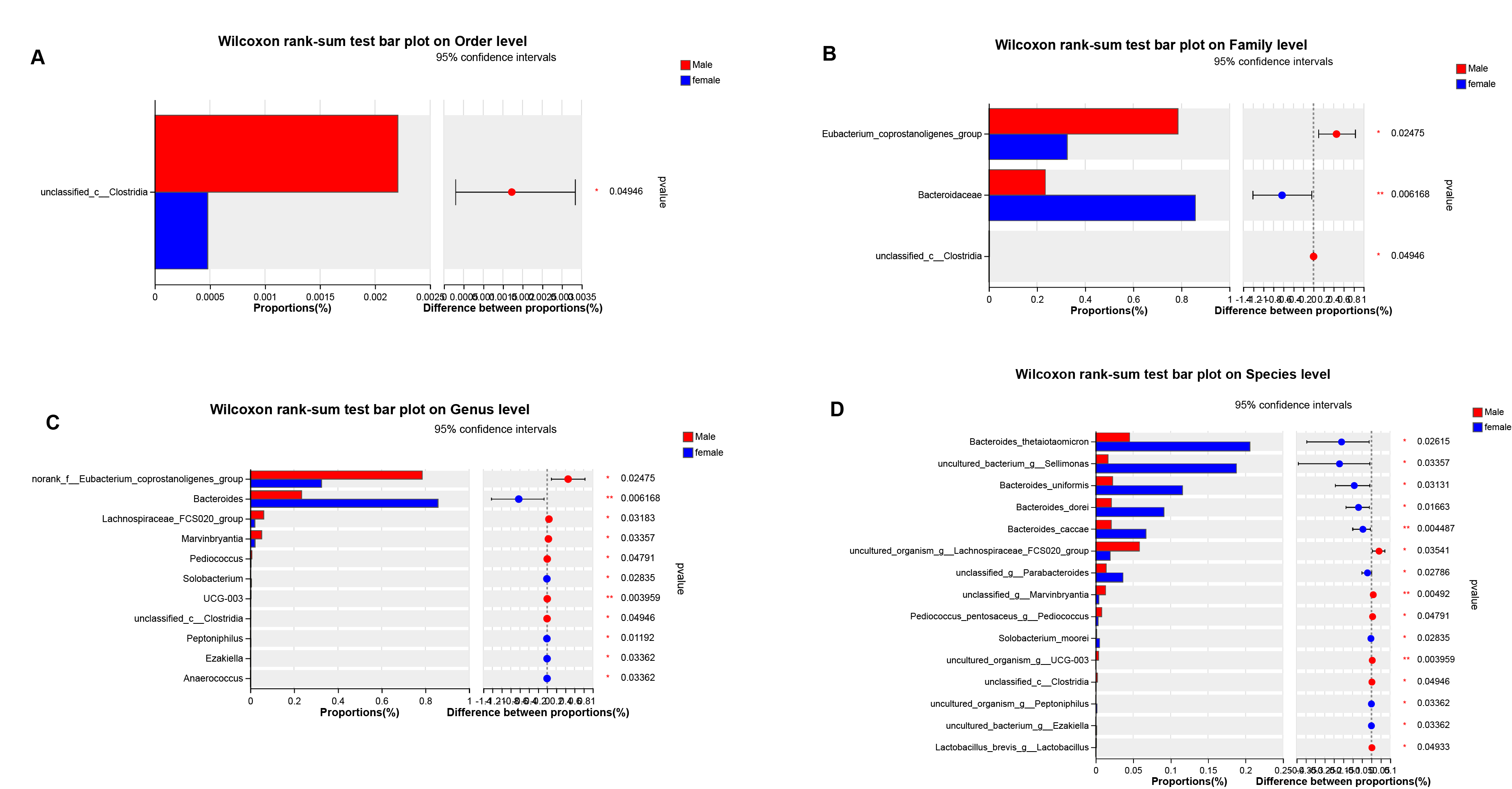

Supplement: Supplementary Figure 1 — TLEA subgroup analysis. The abundance of bacterial community at order (A), family (B), genus (C), and species (D) levels in different gender. TLEA, temporal lobe epilepsy with anxiety disorders. [file Image_1.tif]

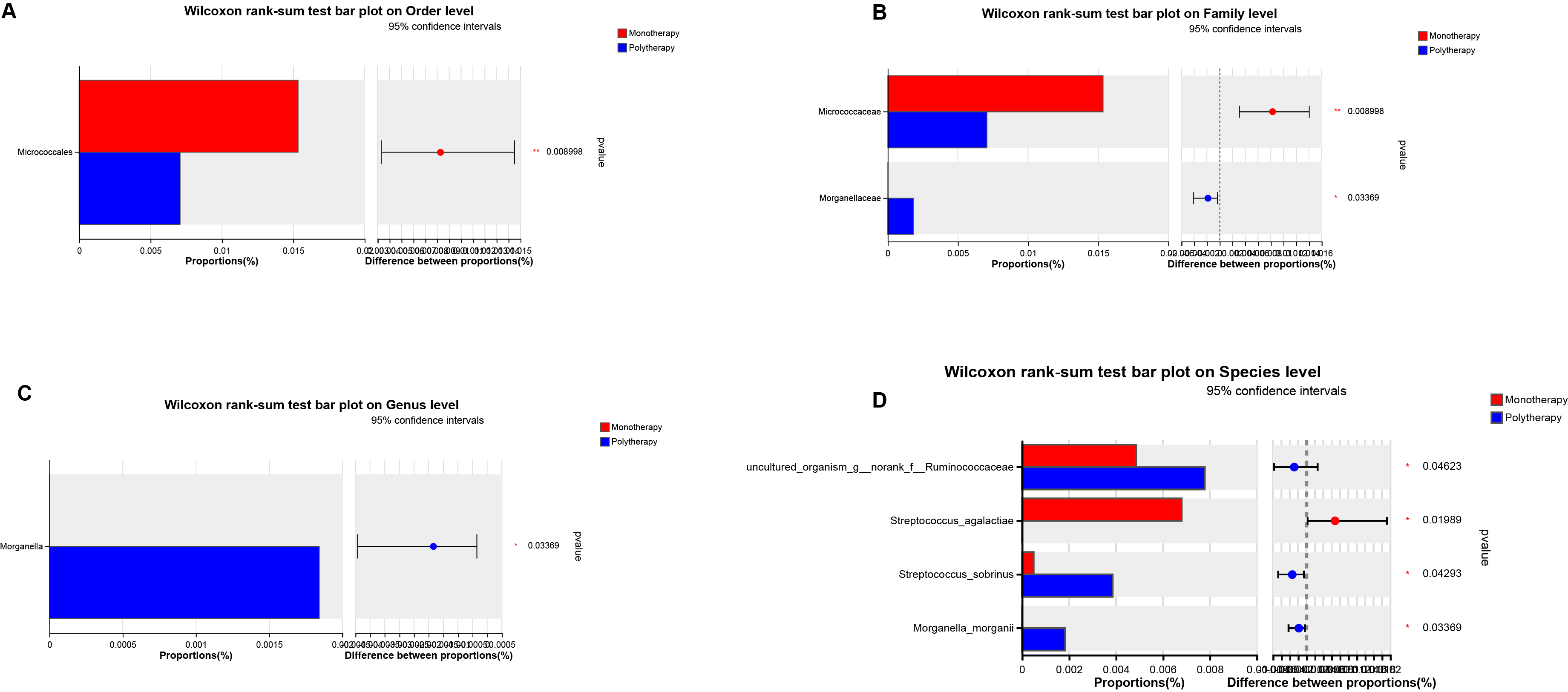

Supplement: Supplementary Figure 2 — TLEA subgroup analysis. The abundance of bacterial community order (A), family (B), genus (C), and species (D) levels in the different treatment regimens. TLEA, temporal lobe epilepsy with anxiety disorders. [file Image_2.tif]

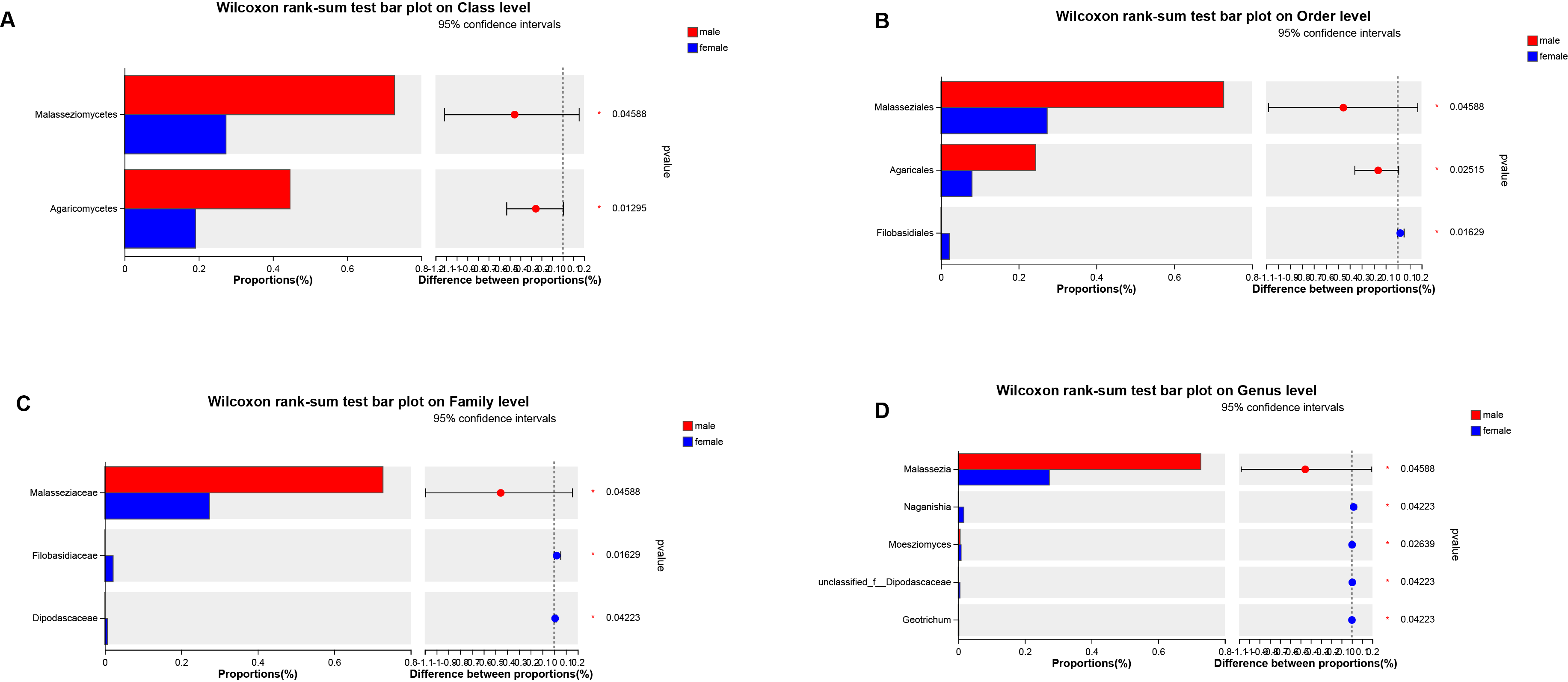

Supplement: Supplementary Figure 3 — TLEA subgroup analysis: the abundance of gender fungal community at class (A), order (B), family (C), and genus (D) levels. TLEA, temporal lobe epilepsy with anxiety disorders. [file Image_3.tif]

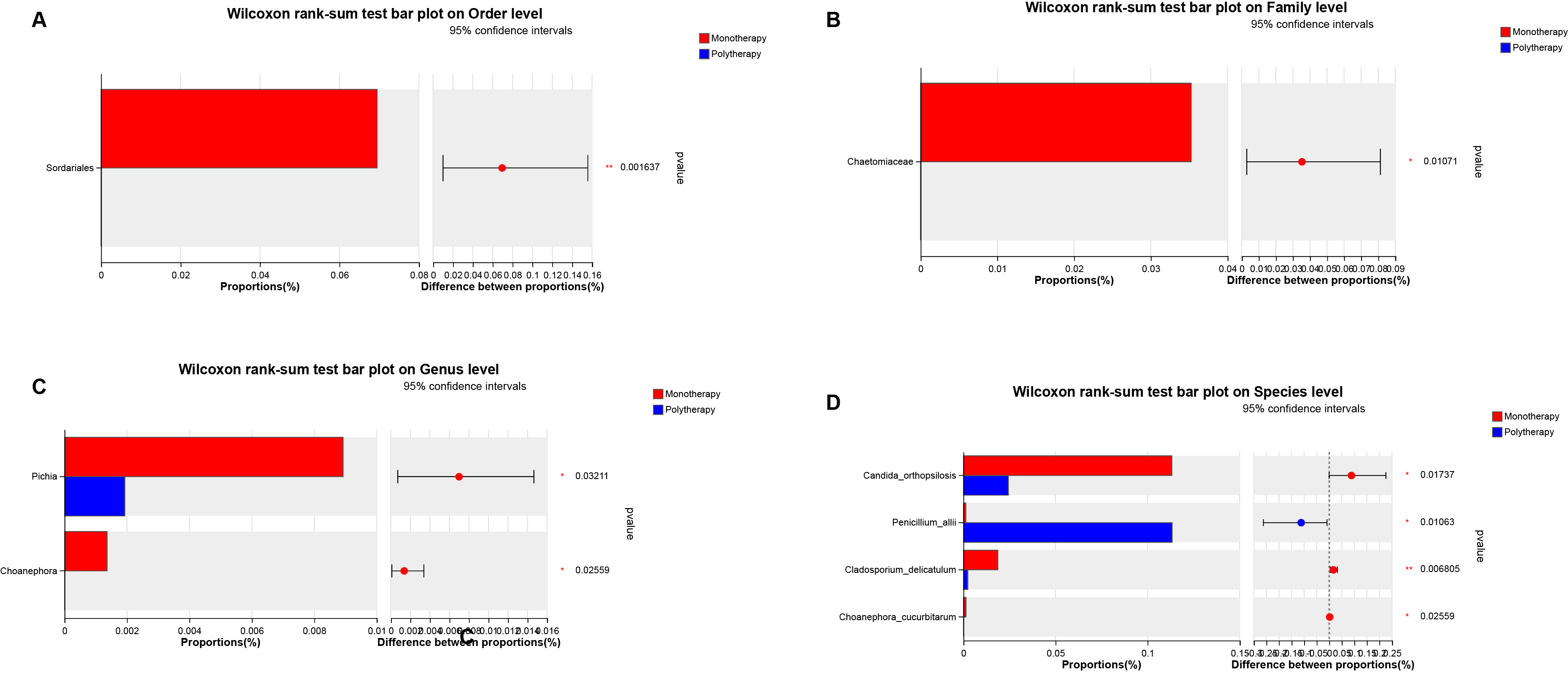

Supplement: Supplementary Figure 4 — TLEA subgroup analysis: the abundance of treatment regimen fungal community at order (A), family (B), genus (C), and species (D) levels. TLEA, temporal lobe epilepsy with anxiety disorders. [file Image_4.tif]
