# Supplementary material for: Altered gut microbiota in temporal lobe epilepsy with anxiety disorders
Source: Front Microbiol. 2023 May 22;14:1165787. doi: 10.3389/fmicb.2023.1165787 (PMC10239838; doi:10.3389/fmicb.2023.1165787)
Supplement: Supplementary Table 1 — Redundancy analysis (RDA) of anxiety impact factors. [file Data_Sheet_1.ZIP › Supplementary Tables/Supplementary Table 3.doc]

**Supplementary Table 3. Redundancy analysis (RDA) of anxiety impact factors**

| **Risk factors** | **RDA1** | **RDA2** | ***r^2^*** | **P values** |
| --- | --- | --- | --- | --- |
| Working strength | -0.1696 | 0.9855 | 0.0596 | 0.29 |
| Hosptialization frequency yearly | 0.9891 | -0.1476 | 0.1956 | 0.014 |
| Treamtment regimen | 0.5532 | -0.833 | 0.1123 | 0.076 |
