# Supplementary material for: Altered gut microbiota in temporal lobe epilepsy with anxiety disorders
Source: Front Microbiol. 2023 May 22;14:1165787. doi: 10.3389/fmicb.2023.1165787 (PMC10239838; doi:10.3389/fmicb.2023.1165787)
Supplement: Supplementary Table 1 — Redundancy analysis (RDA) of anxiety impact factors. [file Data_Sheet_1.ZIP › Supplementary Tables/Supplementary Table 2.doc]

**Supplementary Table 2. Characteristics of 45 temporal lobe epilepsy patients***

| **Characteristics** | **Statistics** | **TLEA**  **n=28** | **TLEW**  **n=17** |  | **P** |
| --- | --- | --- | --- | --- | --- |
| **Age** | yrs, mean ± SD | 29.5 ± 5.7 | 31.1 ±6.8 | 0.828 | 0.413 |
| **BMI** | kg/ m2，mean ± SD | 23.5 ± 1.9 | 22.5 ±2.8 | -1.552 | 0.128 |
| **SAS** | mean ± SD | 59.7± 3.7 | 28.0 ± 3.3 | -29.142 | 0.000 |
| **GAD-7** | mean ± SD | 12.2±1.5 | 1.8 ± 0.9 | -25.237 | 0.000 |
| **HAMA** | mean ± SD | 22.0 ± 4.2 | 5.4 ± 1.3 | -19.344 | 0.000 |
| **Gender** | Female | 16(59.3) | 11(61.1) | 0.252 | 0.616 |
|  | Male | 12(40.7) | 6(38.9) |  |  |
| **Marital status** | Married | 18(63.0) | 10(61.1) | 0.134 | 0.714 |
|  | Unmarried or other marital status | 10(37.0) | 7(38.9) |  |  |
| **Education** | Primary (0-9 years) | 5(18.5) | 7(38.9) | 3.068 | 0.216 |
|  | Secondary (9-12 years) | 17(59.3) | 8(50.0) |  |  |
|  | Higher (12 years) | 6(22.2) | 2(11.1) |  |  |
| **Employment** | Employed（or student） | 15(51.9) | 14(83.3) | 3.824 | 0.051 |
|  | Unemployed | 13(48.1) | 3(16.7) |  |  |
| **Working strength** | Less activity (office, and so on) | 9(29.6) | 4(27.8) | 10.595 | 0.005 |
|  | Light-to-moderate activity (installers and so on) | 9(33.3) | 13(72.2) |  |  |
|  | Moderate or heavy activity (agriculture and so on | 10(37.0) | 0(0) |  |  |
| **Capital income per month (RMB)** | ＜2,000 | 7(25.9) | 7(38.9) | 2.777 | 0.427 |
|  | 2,000-4,999 | 15(51.9) | 9(55.6) |  |  |
|  | 5,000-9,999 | 4(14.8) | 1(12.5) |  |  |
|  | ＞10,000 | 2(7.4) | 0(4.2) |  |  |
| **Type of insurance** | Rural cooperative medical care | 11(37.0) | 10(62.5) | 1.681 | 0.431 |
|  | Urban medical insurance | 13(48.1) | 5(29.2) |  |  |
|  | None | 4(14.8) | 2(8.3) |  |  |
| **Distance to hospital (km)** | ≥10 | 22(77.8) | 9(55.6) | 3.242 | 0.072 |
|  | ＜10 | 6(22.2) | 8(44.4) |  |  |
| **Hospitalization frequency yearly** | 0 | 4(14.8) | 2(11.1) | 9.903 | 0.007 |
|  | 1-4 | 21(77.8) | 6(33.3) |  |  |
|  | ≥5 | 3(7.4) | 9(55.6) |  |  |
| **Medical care** | Very good/good | 15(51.9) | 12(72.2) | 1.276 | 0.259 |
|  | Bad/no idea | 13(48.1) | 5(27.8) |  |  |
| **Beyond annual household income**** | Yes | 14(44.4) | 11(66.7) | 0.926 | 0.336 |
|  | No | 14(55.6) | 6(33.3) |  |  |
| **Convenience of appointment** | Yes | 16(59.3) | 8(44.4) | 0.432 | 0.511 |
|  | No/sometimes | 12(40.7) | 9(55.6) |  |  |
| **Epilepsy syndrome** | Symptomatic focal epilepsy | 14(48.1) | 11(66.7) | 0.926 | 0.336 |
|  | Cryptogenic generalized | 14(51.9) | 6(33.3) |  |  |
| **Treatment regimen** | Monotherapy | 13(42.9) | 14(77.8) | 3.913 | 0.048 |
|  | Polytherapy | 15(57.1) | 3(22.2) |  |  |
| **Seizures in the past 30 days** | Controlled | 12(39.3) | 9(55.6) | 0.432 | 0.511 |
|  | Uncontrolled | 16(60.7) | 8(44.4) |  |  |
| **Current antiepileptic drugs** | Carbamazepine | 8 | 8 | 1.578 | 0.209 |
|  | Oxasipine | 11 | 9 | 0.799 | 0.371 |
|  | Topiramate | 8 | 5 | 0.004 | 0.952 |
|  | Sodium valproate | 9 | 2 | 2.378 | 0.123 |
|  | Pirampanide | 4 | 0 | 2.666 | 0.103 |
|  | Chinese Medicine | 11 | 6 | 0.252 | 0.616 |
|  | Others | 8 | 4 | 0.303 | 0.582 |
| **Disease duration (years)** | ＜0.5 | 7(22.2) | 6(50) | 3.447 | 0.178 |
|  | 0.5-2 | 5(18.5) | 6(25) |  |  |
|  | ＞2 | 16(59.2) | 5(25) |  |  |
| **Daily dosing frequency** | ＜1 | 2(3.7) | 3(22.2) | 5.280 | 0.260 |
|  | 1 | 4(14.8) | 2(11.1) |  |  |
|  | 2 | 1(3.7) | 2(11.1) |  |  |
|  | 3 | 10(37.0) | 8(44.4) |  |  |
|  | ＞3 | 11(40.7) | 2(11.1) |  |  |
| **Perception of seizure control** | Controlled | 7(22.2) | 9(58.3) | 3.604 | 0.058 |
|  | Uncontrolled or not always controlled | 21(77.8) | 8(41.7) |  |  |
| **Care from family or friends** | Continuous/ almost | 24(88.9) | 17(94.4) | 0.305 | 0.581 |
|  | Rare/absent/never | 3(11.1) | 1(5.6) |  |  |

Notes: * Categorical variables were summarized according to the absolute frequency and percentage of subjects (%) in each category level.

** Beyond annual household income, the health care costs exceed the average annual household income.

SAS, Self-Rating Anxiety Scale；GAD, Generalized Anxiety Disorder; HAMA, Hamilton Anxiety Scale
