# Supplementary material for: Altered gut microbiota in temporal lobe epilepsy with anxiety disorders
Source: Front Microbiol. 2023 May 22;14:1165787. doi: 10.3389/fmicb.2023.1165787 (PMC10239838; doi:10.3389/fmicb.2023.1165787)
Supplement: Supplementary Table 1 — Redundancy analysis (RDA) of anxiety impact factors. [file Data_Sheet_1.ZIP › Supplementary Tables/Supplementary Table 1.doc]

**Supplementary Table 1. Redundancy analysis (RDA) of anxiety impact factors**

| **Risk factors** | **RDA1** | **RDA2** | **r2** | **P values** |
| --- | --- | --- | --- | --- |
| Employment | -0.9985 | -0.0554 | 0.1623 | 0.009 |
| Working strength | 0.4813 | -0.8766 | 0.0338 | 0.42 |
| Distance to distance | 0.2706 | 0.9627 | 0.0476 | 0.3 |
| Hosptialization frequency yearly | -0.9684 | -0.2495 | 0.0598 | 0.226 |
| Treamtment regimen | -0.7958 | -0.6056 | 0.1031 | 0.072 |
| Perception_of_seizure_control | -0.8049 | 0.5934 | 0.1555 | 0.019 |
